# Oxytocin receptor neurons in the paraventricular thalamus as a nexus for social behaviour and fear

**DOI:** 10.1093/brain/awaf421

**Published:** 2026-03-20

**Authors:** Kazuhiko Yamamuro, Minobu Ikehara, Yuki Noriyama, Mamiko Okuda, Kazuki Okumura, Kiwamu Matsuoka, Natsuko Kashida, Rio Ishida, Tsutomu Takeda, Michihiro Toritsuka, Tomoko Ochi, Toshiteru Miyasaka, Yumi Tai, Kouko Tatsumi, Tsuyoshi Hattori, Toshihiro Tanaka, Yasuhiko Saito, Nakao Iwata, Manabu Makinodan

**Affiliations:** Center for Health Control, Nara Medical University, Kashihara 634-8521, Japan; Department of Psychiatry, Nara Medical University, Kashihara 634-8521, Japan; Department of Psychiatry, Nara Medical University, Kashihara 634-8521, Japan; Department of Psychiatry, Nara Medical University, Kashihara 634-8521, Japan; Department of Psychiatry, Nara Medical University, Kashihara 634-8521, Japan; Department of Psychiatry, Nara Medical University, Kashihara 634-8521, Japan; Department of Psychiatry, Nara Medical University, Kashihara 634-8521, Japan; Department of Psychiatry, Nara Medical University, Kashihara 634-8521, Japan; Department of Psychiatry, Nara Medical University, Kashihara 634-8521, Japan; Department of Psychiatry, Fujita Health University, Toyoake 470-1192, Japan; Division of Transformative Psychiatry and Synergistic Research, International Center for Brain Science, Fujita Health University, Toyoake 470-1192, Japan; Department of Neuropsychiatry, Kumamoto University, Kumamoto 860-8555, Japan; Department of Psychiatry, Nara Medical University, Kashihara 634-8521, Japan; Department of Psychiatry, Fujita Health University, Toyoake 470-1192, Japan; Division of Transformative Psychiatry and Synergistic Research, International Center for Brain Science, Fujita Health University, Toyoake 470-1192, Japan; Department of Neuropsychiatry, Kumamoto University, Kumamoto 860-8555, Japan; Department of Psychiatry, Nara Medical University, Kashihara 634-8521, Japan; Department of Psychiatry, Fujita Health University, Toyoake 470-1192, Japan; Division of Transformative Psychiatry and Synergistic Research, International Center for Brain Science, Fujita Health University, Toyoake 470-1192, Japan; Department of Neuropsychiatry, Kumamoto University, Kumamoto 860-8555, Japan; Department of Diagnostic and Interventional Radiology, Nara Medical University, Kashihara 634-8521, Japan; Department of Diagnostic and Interventional Radiology, Nara Medical University, Kashihara 634-8521, Japan; Department of Diagnostic and Interventional Radiology, Nara Medical University, Kashihara 634-8521, Japan; Department of Anatomy and Neuroscience, Nara Medical University, Kashihara 634-8521, Japan; Department of Anatomy and Neuroscience, Nara Medical University, Kashihara 634-8521, Japan; Department of Diagnostic and Interventional Radiology, Nara Medical University, Kashihara 634-8521, Japan; Department of Neurophysiology, Nara Medical University, Kashihara 634-8521, Japan; Department of Psychiatry, Fujita Health University, Toyoake 470-1192, Japan; Department of Psychiatry, Nara Medical University, Kashihara 634-8521, Japan; Department of Psychiatry, Fujita Health University, Toyoake 470-1192, Japan; Division of Transformative Psychiatry and Synergistic Research, International Center for Brain Science, Fujita Health University, Toyoake 470-1192, Japan; Department of Neuropsychiatry, Kumamoto University, Kumamoto 860-8555, Japan

**Keywords:** oxytocin, paraventricular thalamus, social behaviour, fear, neural circuits

## Abstract

Oxytocin has been implicated in regulating social behaviour and emotional responses; however, the underlying neural circuits remain incompletely understood. Neurons expressing oxytocin receptors (OTRs) in the paraventricular thalamus (PVT) are emerging as a potential modulator of these processes.

In this study, we investigated the specific role of OTR-expressing PVT neurons in sociability and fear-related behaviours. Using chemogenetic approaches, we found that bidirectional manipulation of these neurons significantly modulated social behaviour and fear extinction in mice. Inhibition of OTR-expressing PVT neurons impaired sociability and fear extinction, whereas activation selectively enhanced early extinction learning without affecting sociability. Electrophysiological analyses revealed that oxytocin increases tonic firing in PVT neurons, suggesting a mechanism for heightened excitability. In contrast, manipulation of OTR-expressing neurons in the medial prefrontal cortex had no effect on sociability. In a complementary human dataset, salivary oxytocin levels were modestly associated with thalamic microstructure and autism spectrum disorder trait severity.

Although the experimental paradigms differed across species, these findings collectively suggest that OTR-expressing PVT neurons may contribute to social and emotional behaviours through circuit-specific mechanisms. These findings may have implications for psychiatric conditions such as autism spectrum disorder and anxiety. Future translational studies should explore the therapeutic potential of targeting oxytocin-related PVT function to treat social and fear-related deficits. Overall, these findings advance our understanding of the role of oxytocin in brain function and its relevance to mental health.

## Teaser

Yamamuro *et al.* show that oxytocin receptor-expressing neurons in the paraventricular thalamus (PVT) influence social behaviour and fear extinction in mice. Their findings link oxytocin signalling, PVT function, and autism spectrum-related traits, highlighting the PVT as a potential therapeutic target for social and fear-related disorders.

## Introduction

Social and fear dysfunctions are common features of psychiatric and neurodevelopmental conditions such as mood disorders, anxiety and autism spectrum disorder (ASD).^1,2^ In addition to core social deficits, individuals with ASD frequently exhibit heightened threat responses, impaired fear extinction and trauma-like symptoms that overlap with post-traumatic stress disorder.^3,4^ These observations suggest that dysregulation of fear circuits may contribute to ASD comorbidities, particularly in the context of social withdrawal and anxiety. Although oxytocin-based interventions can reduce social avoidance in some individuals, conclusions regarding their therapeutic efficacy remain inconsistent.^5-8^ Oxytocin reportedly promotes affiliative behaviours and enhances social interactions in certain contexts when administered intranasally.^5,6,9,10^ However, its effects are context dependent and often vary, particularly under stress or perceived threat.^7,8^ Oxytocin receptors (OTRs) have been identified in the paraventricular thalamus (PVT), a region previously implicated in feeding and maternal behaviours.^11-15^ However, the specific contribution of OTR-expressing PVT neurons to the integration of social behaviour and fear memory remains poorly understood.

The PVT also regulates arousal,^16^ stress,^17,18^ fear,^19,20^ appetitive learning,^21^ incentive salience,^22^ relapse to drug-seeking,^23^ social behaviours^24^ and feeding.^25^ Its involvement in social behaviours is well documented. Experimental evidence suggests that PVT manipulation significantly influences reward- and fear-related behaviours, particularly through its interaction with other brain regions.^26,27^ Our previous study demonstrated that juvenile social isolation disrupts medial prefrontal cortex (mPFC)-PVT circuitry, leading to impairments in social behaviours.^24,28^ However, the specific role of OTRs in the PVT and how they influence the interplay between social behaviour and fear memory remains unclear. Although the PVT displays some anatomical and functional similarities between mice and humans,^29^ oxytocin has been implicated in the modulation of social behaviour and fear responses across species.^30^ Studies investigating OTRs in the mouse PVT suggest that oxytocin may facilitate sociability and attenuate anxiety-like behaviour under certain conditions,^30^ suggesting that studying oxytocin-related PVT function could be beneficial for understanding and treating social and emotional deficits in humans.

The aim of this study was to examine the potential contribution of OTR-expressing neurons to social behaviour and fear regulation using *Oxtr^tm1(cre/GFP)Rpa^*/J (*Oxtr*-Cre) mice and Cre-dependent designer receptors exclusively activated by designer drugs (DREADDs). To this end, we employed a multifaceted approach, combining slice electrophysiology and viral vector-based chemogenetic manipulation of OTR-expressing neurons to investigate their potential involvement in sociability and fear-related behaviours.

## Materials and methods

Detailed methodological information is provided in the Supplementary material.

### Animals

Male C57BL/6J wild-type mice and *Oxtr*-Cre mice (stock no. 030543, Jackson Laboratory) were used. Animals were housed under a 12 h light-dark cycle. Viral injections were performed in *Oxtr*-Cre mice at 6–7 weeks of age for behavioural experiments and at 9–12 weeks for electrophysiological studies. Behavioural testing was conducted when mice were 10–15 weeks old (each weighing approximately 30 g). All experiments were approved by the Animal Care Committee of Nara Medical University and conducted in accordance with its guidelines, and the ARRIVE (Animal Research: Reporting of *In Vivo* Experiments) guidelines (https://arriveguidelines.org).

### Human assessment of autism symptoms and salivary data

Studies involving human participants were reviewed and approved by the Nara Medical University Ethics Committee (Approval Number: 1319, 2535) and all participants provided written informed consent prior to inclusion. We recruited 19 individuals with ASD (mean age: 14.526 ± 1.264 years, four females) and 11 typically developing (TD) participants (mean age: 14.270 ± 1.348 years, three females). All participants were Japanese and resided in Japan. Patients with ASD were recruited from the out-patient psychiatry services at Nara Medical University Hospital and its affiliated clinics. ASD diagnoses were made by two trained psychiatrists according to the Diagnostic and Statistical Manual of Mental Disorders, 5th edition (DSM-5), and further validated with the Autism Diagnostic Observation Schedule-2 (ADOS-2).^31^ Cognitive function was assessed using the Das-Naglieri Cognitive Assessment System (DN-CAS), a theory-driven measure based on the Planning, Attention, Simultaneous and Successive (PASS) theory.^32^ The PASS theory conceptualizes intelligence as a process-oriented framework consisting of four cognitive processes: planning, attention, simultaneous processing and successive processing. Participants scoring below 70 on the total DN-CAS scale were excluded. Individuals diagnosed with other neurological and psychiatric disorders were also excluded. All participants completed the Japanese version of the Autism-Spectrum Quotient (AQ-J)^33^ and the 30-item General Health Questionnaire (GHQ-30).^34,35^ In addition, saliva samples were collected from all participants to measure oxytocin concentrations for subsequent analyses.

### Statistical analyses

All statistical analyses were performed using Prism version 9 (GraphPad Software Inc., San Diego, CA, USA). Sample sizes were determined based on previous studies.^24,36^ For normally distributed data with equal variances (as assessed by the Shapiro–Wilk test and the F-test), between-group differences were analysed using unpaired two-tailed Student’s *t*-tests. For non-normally distributed data, as assessed by the Shapiro–Wilk test, the nonparametric Mann–Whitney U-test was used. Right-skewed data were log-transformed before statistical analyses. Two-way ANOVA followed by Bonferroni *post hoc* tests were used to analyse the time spent in each zone of the three-chamber social preference test.^24,36^ Two-way repeated-measures ANOVA was used to assess the cumulative number of contacts in the augmented reality-based long-term animal behaviour observing system (AR-LABO) task.

Between-group differences in age, the neurite density index (NDI), orientation dispersion index (ODI), AQ-J total and subscale scores, and GHQ-30 total and subscale scores were examined using unpaired two-tailed Student’s *t*-tests (*P* < 0.05). Fisher’s exact test was employed to examine sex distribution differences (*P* < 0.05). Multiple linear stepwise regression analyses were conducted to identify independent predictors of salivary oxytocin levels, with NDI [thalamus and dorsolateral prefrontal cortex (DLPFC)] and ODI (thalamus and DLPFC) as independent variables for both TD and ASD groups. Additional multiple linear stepwise regression analyses were performed to examine predictors of AQ-J total score and its five subscale scores (social skills, attention switching, local details, communication and imagination), as well as the six subscales of the GHQ-30 (general disease trends, physical conditions, sleeping disorder, social anxiety disorder, anxiety and dysthymia, suicidal ideation and depression), using NDI and ODI measures as independent variables. Differences in the proportions of excitatory and inhibitory OTR-expressing neurons between the PVT and mPFC were analysed using a chi-square test. All data are presented as mean ± standard error of the mean. A *P*-value <0.05 was considered statistically significant.

## Results

### Suppressing the activity of OTR-expressing PVT neurons, but not mPFC neurons, reduces sociability

**Figure 1:**
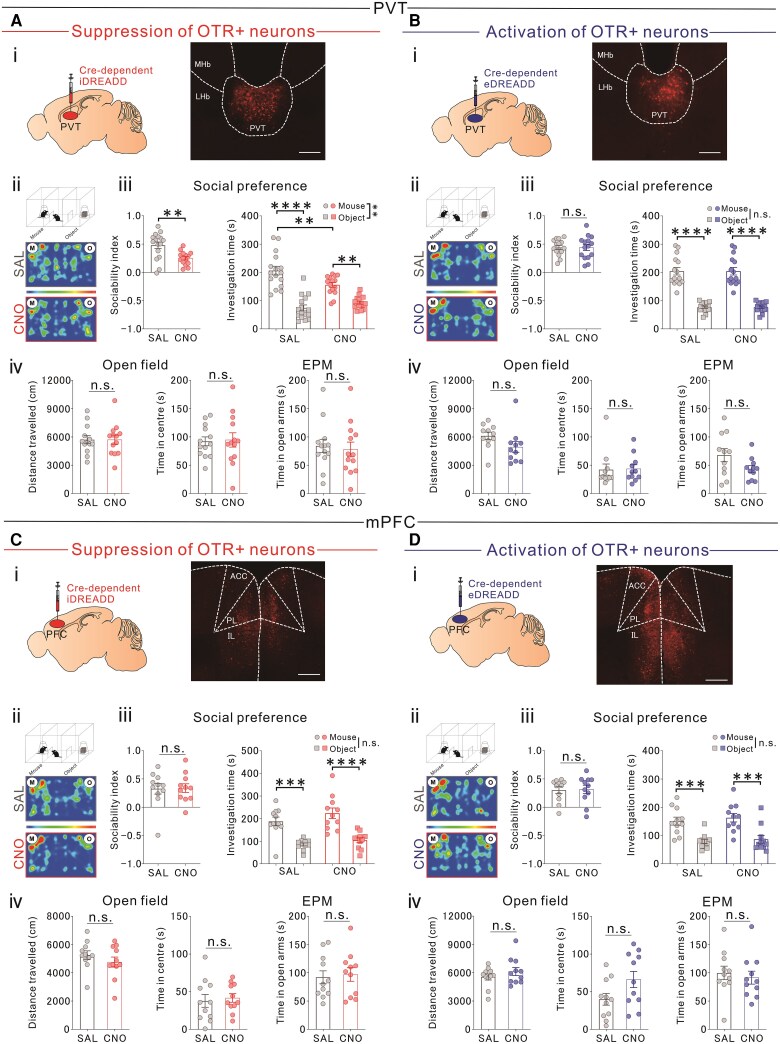
**Chemogenetic suppression of OTR-expressing PVT neurons reduces sociability in adult mice.** [**A**(**i**)] *Left*: Cre-dependent iDREADD was injected into the PVT of *Oxtr*-Cre mice to express iDREADD in OTR-expressing neurons. *Right*: Representative PVT images showing iDREADD-mCherry expression. Scale bar = 500 μm. [**A**(**ii**)] iDREADD+ mice received intraperitoneal SAL/CNO (5 mg/kg) before a three-chamber sociability test. Representative heat maps show the movement patterns of SAL- and CNO-treated mice during the test. [**A**(**iii**)] CNO-treated iDREADD+ mice exhibited reduced sociability (two-tailed *t*-test, *t*_29_ = 3.403, **P = 0.0020), confirmed by three-chamber test [two-way RM ANOVA, Drug (CNO/SAL) × Stimulus (social/object) interaction, *F*(1,58) = 10.060, **P = 0.0024; drug effect *F*(1,58) = 22.118, *P =* 0.1510; stimulus effect *F*(1,58) = 80.810, ****P < 0.0001, Bonferroni *post hoc*: social versus object exposure in SAL ****P < 0.0001, social versus object exposure in CNO ***P = 0.0003, CNO versus SAL in social exposure **P = 0.0036; (SAL: *n* = 16, CNO: *n* = 15)]. [**A**(**iv**)] No differences in motor activity or anxiety-related behaviour (open-field distance travelled, two-tailed unpaired *t*-test, *t*_24_ = 0.003, *P =* 0.998; open-field time in centre: *t*_24_ = 0.169, *P =* 0.8670; EPM time in open arms: *t*_24_ = 0.169, *P =* 0.7256; SAL: *n* = 13, CNO: *n* = 13). [**B**(**i**)] *Left*: Cre-dependent eDREADD was injected into the PVT. *Right*: Representative PVT images of eDREADD-mCherry expression. Scale bar = 500 μm. [**B**(**ii**)] eDREADD+ mice received SAL or CNO (1 mg/kg) before sociability testing. Representative heat maps show the movement patterns of SAL- and CNO-treated mice. [**B**(**iii**)] CNO-treated eDREADD+ mice showed no sociability differences (two-tailed *t*-test, *t*_28_ = 0.030, *P =* 0.9760; SAL: *n* = 15, CNO: *n* = 15) nor differences in investigation time [two-way RM ANOVA, Drug × Stimulus interaction, *F*(1,58) = 0.567, *P =* 0.454; drug effect *F*(1,56) = 2.331, *P =* 0.1324; stimulus effect *F*(1,56) = 158.400, ****P < 0.0001, Bonferroni *post hoc*: social versus object exposure in SAL ****P < 0.0001, social versus object exposure in CNO ****P < 0.0001]. [**B**(**iv**)] No differences in motor activity or anxiety-related behaviour (open-field distance travelled, two-tailed unpaired *t*-test, *t*_20_ = 1.721, *P =* 0.1006; open-field time in centre: *t*_20_ = 0.122, *P =* 0.9040; EPM time in open centre: *t*_20_ = 1.879, *P =* 0.0748; SAL: *n* = 11, CNO: *n* = 11). [**C**(**i**)] *Left*: Cre-dependent iDREADD was injected into the mPFC. *Right*: mPFC images of iDREADD-mCherry. Scale bar = 500 μm. [**C**(**ii**)] iDREADD+ mice received SAL or CNO (5 mg/kg) before sociability testing. Representative heat maps show movement patterns of SAL- and CNO-treated mice. [**C**(**iii**)] No sociability differences were observed in CNO-treated iDREADD+ mice (two-tailed *t*-test, *t*_20_ = 0.097, *P =* 0.9239), nor differences in investigation time [two-way RM ANOVA, Drug × Stimulus interaction, *F*(1,40) = 0.262, *P =* 0.6113; drug effect *F*(1,40) = 2.824, *P =* 0.1007; stimulus effect *F*(1,40) = 45.940, ****P < 0.0001, Bonferroni *post hoc*: social versus object exposure in SAL ***P = 0.0001, social versus object exposure in CNO ****P < 0.0001]. [**C**(**iv**)] No differences in motor activity or anxiety-related behaviour (open-field distance travelled, two-tailed unpaired *t*-test, *t*_20_ = 1.066, *P =* 0.2989; open-field time in the centre: *t*_20_ = 0.378, *P =* 0.7093; EPM time in open arms: *t*_20_ = 0.298, *P =* 0.7688; SAL: *n* = 11, CNO: *n* = 11). [**D**(**i**)] *Left*: Cre-dependent eDREADD was injected into the mPFC. *Right*: mPFC images of eDREADD-mCherry expression. Scale bar = 500 μm. [**D**(**ii**)] eDREADD+ mice received SAL or CNO (1 mg/kg) before sociability testing. Representative heat maps show the movement patterns of SAL- and CNO-treated mice. [**D**(**iii**)] No sociability differences in CNO-treated eDREADD+ mice (two-tailed *t*-test, *t*_20_ = 0.117, *P =* 0.9079) or investigation time difference [two-way RM ANOVA, Drug × Stimulus interaction, *F*(1,40) = 0.028, *P =* 0.8669; drug effect *F*(1,40) = 0.584, *P =* 0.4493; stimulus effect *F*(1,40) = 32.650, ****P < 0.0001, Bonferroni *post hoc*: social versus object exposure in SAL ***P = 0.0007, social versus object exposure in CNO ***P = 0.0003; (SAL: *n* = 11, CNO: *n* = 11)]. [**D**(**iv**)] No significant differences in motor activity or anxiety-related behaviour (open-field distance travelled, two-tailed unpaired *t*-test, *t*_20_ = 1.073, *P =* 0.2961; open-field time in the centre: *t*_20_ = 1.989, *P =* 0.0605; EPM time in open arms: *t*_20_ = 0.502, *P =* 0.6210; SAL: *n* = 11, CNO: *n* = 11). iDREADD = inhibitory designer receptor exclusively activated by designer drugs; eDREADD = excitatory DREADD; PVT = paraventricular thalamus; mPFC = medial prefrontal cortex; OTR = oxytocin receptor; SAL = saline; CNO = clozapine *N*-oxide; EPM = elevated plus maze; PL = prelimbic; IL = infralimbic; MHb = medial habenula; LHb = lateral habenula; RM ANOVA = repeated-measures ANOVA.

To investigate how OTR-expressing neurons in the PVT and mPFC differentially affect sociability, we performed chemogenetic experiments in both regions (Supplementary Figs 1A and B and 2A and B). Although previous findings indicate that PVT suppression impairs social behaviour,^24^ the specific role of OTR-expressing PVT neurons remains unclear. To investigate this, we employed chemogenetic techniques in *Oxtr*-Cre mice to selectively modulate OTR-expressing neuron activity and assess their involvement in sociability. Inhibitory DREADD (iDREADD) expression in OTR-expressing neurons [Fig. 1A(i)], followed by administration of clozapine *N*-oxide dihydrochloride (CNO), significantly reduced sociability in the three-chamber test [Fig. 1A(ii and iii)], without affecting locomotor activity or anxiety-related behaviours [Fig. 1A(iv)]. Conversely, excitatory DREADD (eDREADD)-mediated activation [Fig. 1B(i)] had no significant impact on sociability, motor function or anxiety-like behaviour [Fig. 1B(ii–iv)]. Control experiments involving CNO administration in mCherry-expressing PVT neurons revealed no behavioural changes (Supplementary Fig. 3A–E).

Given the lack of significant effects on sociability following OTR-expressing mPFC neuron manipulation,^37^ we chemogenetically suppressed mPFC neuronal activity in *Oxtr*-Cre mice using iDREADD [Fig. 1C(i)]. CNO administration did not affect sociability [Fig. 1C(ii and iii)], motor activity or anxiety-related behaviours [Fig. 1C(iv)]. Similarly, chemogenetic activation of OTR-expressing mPFC neurons via eDREADD [Fig. 1D(i)] failed to produce any significant impact on sociability [Fig. 1D(ii and iii)], motor activity or anxiety-related behaviour [Fig. 1D(iv) and Supplementary Fig. 3F–J].

### Cell-type characterization of OTR-expressing neurons in the PVT and mPFC

To determine the neuronal phenotype of recombined OTR-expressing neurons, we performed immunohistochemistry. In the PVT, 96% of the 108 OTR-expressing neurons were vGluT1/2+, indicating a predominantly excitatory population with minimal inhibitory involvement (4% GAD65/67+). In contrast, in the mPFC, co-labelling with Cux1 or Ctip2 and GAD65/67 revealed a more heterogeneous population: approximately 75% of the OTR-expressing neurons were excitatory and 25% inhibitory across two independent datasets (Supplementary Figs 4 and 5). These findings confirm that although the OTR-expressing neurons were mainly excitatory in both regions, inhibitory neurons were more prevalent in the mPFC than in the PVT.

### OTR-expressing PVT neurons bidirectionally modulate sociability in free-moving conditions

**Figure 2:**
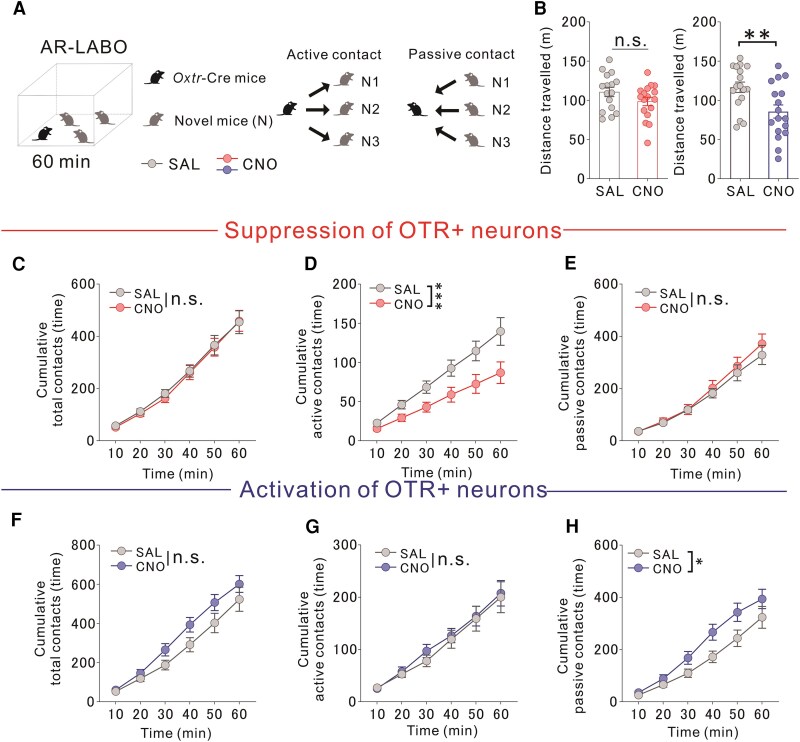
**Chemogenetic control of OTR-expressing PVT neurons bidirectionally affects social behaviour in free-moving mice.** Mice were allowed to freely interact for 60 min with three unfamiliar cage-mates in a novel environment. (**A**) Cre-dependent iDREADD or eDREADD was injected into the PVT of *Oxtr*-Cre mice. Mice received intraperitoneal SAL/CNO (iDREADD+ mice: 5 mg/kg, eDREADD+ mice: 1 mg/kg) and freely interacted with three novel mice for 60 min. Graph: cumulative approaches per 10-min bin. (**B**) *Left*: No difference in distance travelled between CNO-treated iDREADD+ mice and SAL-treated iDREADD+ versus SAL controls (*t*_31_ = 1.522, *P* = 0.1382; SAL: *n* = 16, CNO: *n* = 17). *Right*: CNO-treated eDREADD+ mice travelled less than SAL controls (*t*_32_ = 2.878, **P = 0.0070). (**C**) No difference in cumulative total contact [two-way RM ANOVA, Drug × Time interaction, *F*(5,160) = 0.085, *P =* 0.9945; drug effect *F*(1,32) = 0.059, *P =* 0.8095; time effect *F*(1.278,40.90) = 160.300, ****P < 0.0001; SAL: *n* = 17, CNO: *n* = 17]. (**D**) CNO-treated iDREADD+ mice exhibited fewer active approaches than SAL controls [two-way RM ANOVA, Drug × Time interaction, *F*(5,160) = 4.382, ***P = 0.0009; drug effect *F*(1,32) = 6.264, *P =* 0.0176; time effect *F*(1.162,37.19) = 78.250, ****P < 0.0001; SAL: *n* = 16, CNO: *n* = 17)]. (**E**) No difference in passive approaches [two-way RM ANOVA, Drug × Time interaction, *F*(5,160) = 0.620, *P =* 0.6851; drug effect *F*(1,32) = 0.336, *P =* 0.5664; time effect *F*(1.368,43.77) = 127.700, ****P < 0.0001; SAL: *n* = 16, CNO: *n* = 17]. (**F**) No difference in cumulative total contact for CNO-treated eDREADD+ mice [two-way RM ANOVA, Drug × Time interaction, *F*(5,160) = 1.735, *P =* 0.1294; drug effect *F*(1,32) = 2.591, *P =* 0.1173; time effect *F*(1.431,45.78) = 165.500, ****P < 0.0001; SAL: *n* = 17, CNO: *n* = 17]. (**G**) No difference in active approaches [two-way RM ANOVA, Drug × Time interaction, *F*(5,160) = 0.198, *P =* 0.9630; drug effect *F*(1,32) = 0.135, *P =* 0.7160; time effect *F*(1.483,47.45) = 77.4700, ****P < 0.0001; SAL: *n* = 17, CNO: *n* = 17]. (**H**) CNO-treated eDREADD+ mice exhibited more passive approaches compared with SAL controls [two-way RM ANOVA, Drug × Time interaction, *F*(5,160) = 2.526, *P =* 0.3128; drug effect *F*(1,32) = 3.979, *P =* 0.0546; time effect *F*(1.389,44.45) = 78.250, ****P < 0.0001; SAL: *n* = 17, CNO: *n* = 17]. iDREADD = inhibitory designer receptors exclusively activated by designer drugs; eDREADD = excitatory DREADD; SAL = saline; CNO = clozapine *N*-oxide dihydrochloride; RM ANOVA = repeated-measures analysis of variance; OTR = oxytocin receptor; PVT = paraventricular thalamus.

We evaluated naturalistic social interactions utilizing the augmented reality-based long-term animal behaviour observing system (AR-LABO).^36^ At 2 months of age, experimental mice were introduced into a new cage with three age-matched male C57BL/6J mice who were unfamiliar to the test mouse, and their locomotor activity was recorded for 1 h (Fig. 2A). ‘Active contact’ was defined as contact initiated by the approaching mouse, whereas ‘passive contact’ referred to contact experienced by the receiving mouse (Fig. 2A). Chemogenetic suppression of OTR-expressing PVT neurons did not significantly alter locomotor activity, whereas activation of these neurons reduced locomotor activity compared with control mice (Fig. 2B). Suppressing OTR-expressing PVT neurons in *Oxtr*-Cre mice via CNO treatment reduced active contact frequency without affecting the duration of contact compared with saline (SAL) treatment (Fig. 2C–E and Supplementary Fig. 6A–C). Conversely, activation increased passive contact behaviour significantly without altering contact duration relative to SAL treatment (Fig. 2F–H and Supplementary Fig. 6D–F). Suppression of OTR-expressing PVT neuronal activity also induced ‘stick-to-the-same-mouse’ behaviour, whereas activation did not (Supplementary Fig. 7A–F). To assess the diversity of social engagement among the three unfamiliar partners, we calculated a switching ratio, defined as the number of alternations between different partners divided by the total number of contact bouts. No significant differences were observed across groups (Supplementary Fig. 8). These findings align with the three-chamber test results, where suppression of PVT neurons reduced active contact behaviour. However, the increase in passive contact behaviour was not assessed in the three-chamber test.

### Manipulation of OTR-expressing PVT neurons, not mPFC, affects fear extinction

**Figure 3:**
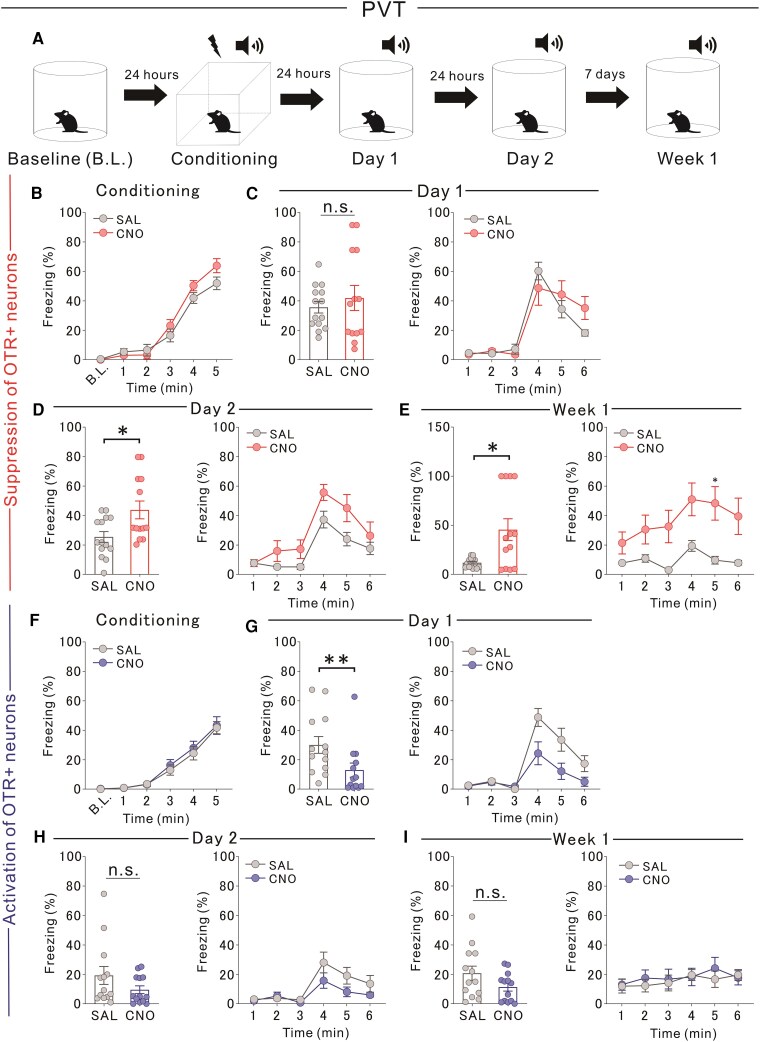
**Chemogenetic manipulations of PVT modulate fear cue extinction learning**. (**A**) Experimental design: Cre-dependent iDREADD/eDREADD vectors were injected into the PVT of *Oxtr*-Cre mice, targeting OTR-expressing neurons. SAL or CNO (iDREADD: 5 mg/kg, eDREADD: 1 mg/kg) was administered 30 min before tests on Day 1, Day 2 and Week 1. (**B**) No difference in fear conditioning between iDREADD+ CNO-treated mice and SAL controls [two-way RM ANOVA, Drug (CNO/SAL) × Time (10-min bins) interaction, *F*(5,125) = 1.523, *P =* 0.1872; drug effect *F*(1,25) = 0.647, *P =* 0.4289; time effect *F*(2.644,66.11) = 167.100, ****P < 0.0001: SAL: *n* = 14, CNO: *n* = 13]. (**C**) No difference in freezing ratio on Day 1 [*Left*: Kolmogorov–Smirnov test, D = 0.319, *P =* 0.5003; *Right*: two-way RM ANOVA, Drug × Time interaction, *F*(5,125) = 2.335, *P = 0.0459; drug effect *F*(1,25) = 0.179, *P =* 0.6757; time effect *F*(1.915,47.87) = 41.060, ****P < 0.0001: SAL: *n* = 14, CNO: *n* = 13]. (**D**) Increased freezing on Day 2 in iDREADD+ CNO-treated mice [*Left*: Mann–Whitney U-test, U = 50, *P = 0.0465; *Right*: two-way RM ANOVA, Drug (CNO or SAL) × Time interaction, *F*(5,125) = 1.433, *P =* 0.2171; drug effect *F*(1,25) = 5.112, *P = 0.0327; time effect *F*(3.197,79.91) = 24.730, ****P < 0.0001: SAL: *n* = 14, CNO: *n* = 13]. (**E**) Increased freezing in iDREADD+ CNO-treated mice at Week 1 [*Left*: Kolmogorov–Smirnov test, D = 0.692, **P = 0.0031; *Right*: two-way RM ANOVA, Drug × Time interaction, *F*(5,125) = 3.413, **P = 0.0063; drug effect *F*(1,25) = 8.327, **P = 0.0079; time effect *F*(3.433,85.83) = 9.451, ****P < 0.0001, Bonferroni *post hoc*: SAL versus CNO in session 5 *P = 0.0326: SAL: *n* = 14, CNO: *n* = 13]. (**F**) No difference in fear conditioning in eDREADD+ CNO-treated mice [two-way RM ANOVA, Drug × Time interaction, *F*(5,125) = 0.203, *P =* 0.9606; drug effect *F*(1,25) = 0.211, *P =* 0.650; time effect *F*(1.858,46.45) = 81.470, ****P < 0.0001: SAL: *n* = 13, CNO: *n* = 13]. (**G**) Reduced freezing on Day 1 in eDREADD+ CNO-treated mice [*Left*: Mann–Whitney U-test, U = 32, **P = 0.0058; *Right*: one-way RM ANOVA, Drug × Time interaction, *F*(5,125) = 4.542, ***P = 0.0008; drug effect *F*(1,25) = 5.868, *P = 0.023; time effect *F*(2.185,54.62) = 26.900, ****P < 0.0001: SAL: *n* = 13, CNO: *n* = 13]. (**H**) Increased freezing on Day 2 in eDREADD+ CNO-treated mice [*Left*: Mann–Whitney U-test, U = 57, *P =* 0.1647; *Right*: two-way RM ANOVA, Drug (CNO or SAL) × Time interaction, *F*(5,125) = 1.791, *P =* 0.1194; drug effect *F*(1,25) = 2.181, *P =* 0.1522; time effect *F*(2.150,53.75) = 13.020, ****P < 0.0001: SAL: *n* = 13, CNO: *n* = 13]. (**I**) No difference in freezing at Week 1 in eDREADD+ CNO-treated mice [*Left*: two-tailed unpaired *t*-test, *t*_24_ = 1.703, *P =* 0.1014; *Right*: two-way RM ANOVA, Drug × Time interaction, *F*(5,125) = 0.635, *P =* 0.6731; drug effect *F*(1,25)= 0.127, *P =* 0.7242; time effect *F*(2.890,72.26) = 1.6790, *P =* 0.1808: SAL: *n* = 13, CNO: *n* = 13]. iDREADD = inhibitory designer receptors exclusively activated by designer drugs; eDREADD = excitatory DREADD; SAL = saline; CNO = clozapine *N*-oxide dihydrochloride; RM ANOVA = repeated-measures ANOVA; PVT = paraventricular thalamus; OTR = oxytocin receptor.

**Figure 4:**
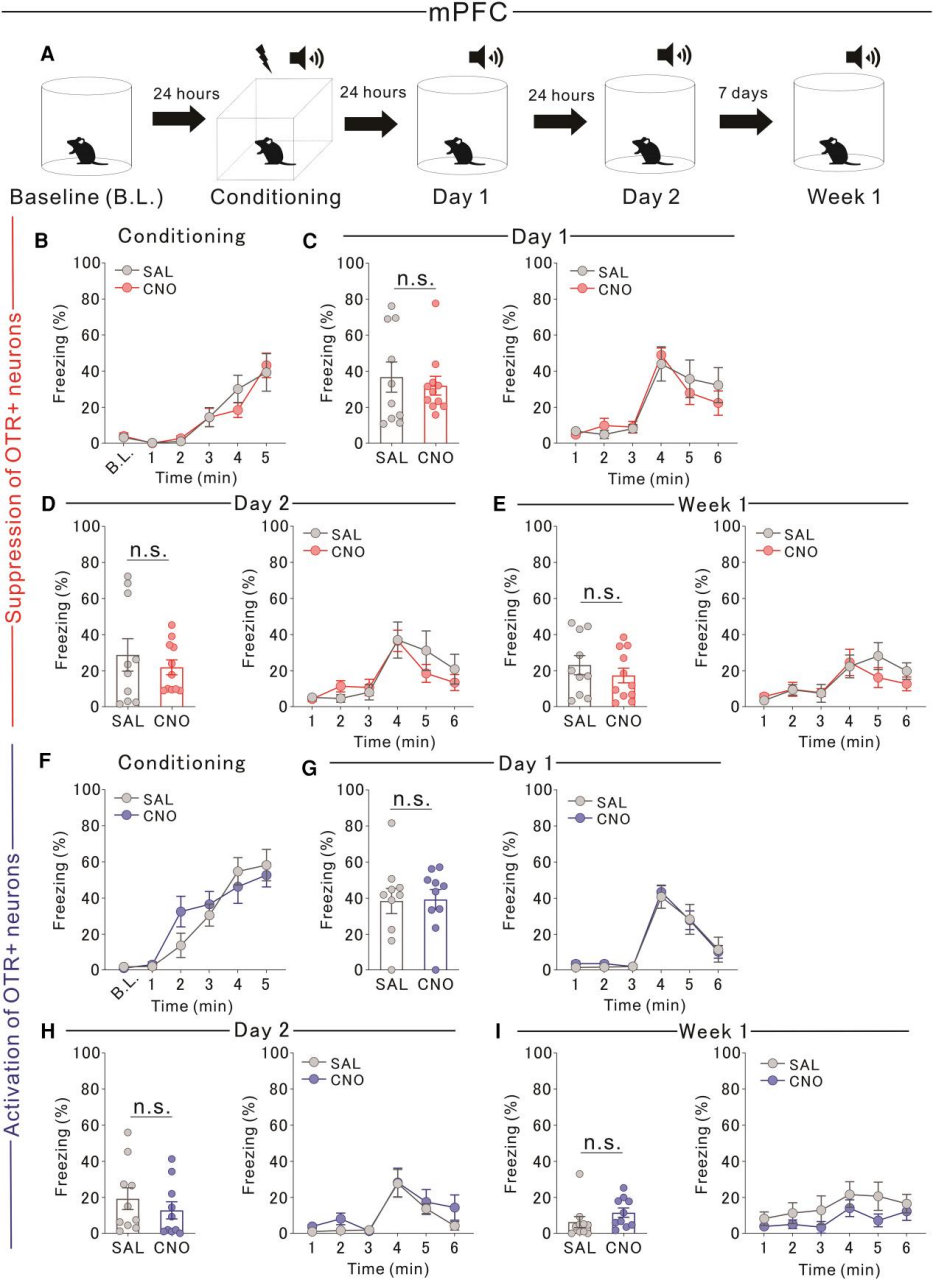
**Chemogenetic manipulation of mPFC does not affect cue-conditioned fear extinction**. (**A**) Experimental design: Cre-dependent iDREADD or eDREADD vectors were injected into the mPFC of *Oxtr*-Cre mice. Mice received SAL or CNO (iDREADD+: 5 mg/kg, eDREADD+: 1 mg/kg) 30 min before tests on Day 1, Day 2 and Week 1. SAL: *n* = 10, CNO: *n* = 10/11. (**B**) No difference in fear conditioning in iDREADD+ CNO-treated mice [two-way RM ANOVA, Drug × Time interaction, *F*(5,95) = 0.823, *P* = 0.5363; drug effect *F*(1,19) = 0.053, *P* = 0.8205; time effect *F*(2.130,40.47) = 28.72, ****P < 0.0001; SAL: *n* = 10, CNO: *n* = 11]. (**C**) No difference in freezing on Day 1 in iDREADD+ CNO-treated mice [*Left*: Mann–Whitney U-test, U = 51, *P* = 0.7951; *Right*: two-way RM ANOVA, Drug × Time interaction, *F*(5,95) = 0.854, *P* = 0.5189; drug effect *F*(1,19) = 0.071, *P* = 0.7931; time effect *F*(2.793,53.08) = 23.460, ****P < 0.0001; SAL: *n* = 10, CNO: *n* = 11]. (**D**) No difference in freezing on Day 2 in iDREADD+ CNO-treated mice [*Left*: Mann–Whitney U-test, U = 52, *P* = 0.8485; *Right*: two-way RM ANOVA, Drug × Time interaction, *F*(5,95) = 1.328, *P* = 0.2592; drug effect *F*(1,19) = 0.108, *P* = 0.7458; time effect *F*(2.360,44.84) = 16.150, ****P < 0.0001; SAL: *n* = 10, CNO: *n* = 11]. (**E**) No difference in freezing at Week 1 in iDREADD+ CNO-treated mice [*Left*: two-tailed unpaired *t*-test, *t*_19_ = 0.865, *P* = 0.3979; *Right*: two-way RM ANOVA, Drug × Time interaction, *F*(5,95) = 1.037, *P* = 0.4008; drug effect *F*(1,19) = 0.313, *P* = 0.5827; time effect *F*(2.727,51.81) = 7.523, ****P < 0.0001; SAL: *n* = 10, CNO: *n* = 11]. (**F**) No difference in fear conditioning in eDREADD+ CNO-treated mice [two-way RM ANOVA, Drug × Time interaction, *F*(5,90) = 1.689, *P* = 0.1453; drug effect *F*(1,18) = 0.103, *P* = 0.7515; time effect *F*(3.607,64.93) = 38.34, ****P < 0.0001; SAL: *n* = 10, CNO: *n* = 10]. (**G**) No difference in freezing on Day 1 in eDREADD+ CNO-treated mice [*Left*: two-tailed unpaired *t*-test, *t*_18_ = 0.099, *P* = 0.9219; *Right*: two-way RM ANOVA, Drug × Time interaction, *F*(5,90) = 0.090, *P* = 0.9937; drug effect *F*(1,18) = 0.056, *P* = 0.8152; time effect *F*(2.335,44.02) = 39.95, ****P < 0.0001; SAL: *n* = 10, CNO: *n* = 10]. (**H**) No difference in freezing on Day 2 in eDREADD+ CNO-treated mice [*Left:* Mann–Whitney U-test, U = 34, *P* = 0.2392; *Right*: two-way RM ANOVA, Drug × Time interaction, *F*(5,90) = 0.517, *P* = 0.7632; drug effect *F*(1,18) = 0.937, *P* = 0.3455; time effect *F*(2.241,40.34) = 13.500, ****P < 0.0001; SAL: *n* = 10, CNO: *n* = 10]. (**I**) No difference in freezing at Week 1 in eDREADD+ CNO-treated mice [*Left*: Mann–Whitney U-test, U = 25.50, *P* = 0.0655; *Right*: two-way RM ANOVA, Drug × Time interaction, *F*(5,90) = 0.444, *P* = 0.8168; drug effect *F*(1,18) = 1.810, *P* = 0.1952; time effect *F*(2.379,42.83) = 3.061, *P = 0.0489; SAL: *n* = 10, CNO: *n* = 10]. iDREADD = inhibitory designer receptors exclusively activated by designer drugs; eDREADD = excitatory designer receptors exclusively activated by designer drugs; SAL = saline; CNO = clozapine *N*-oxide dihydrochloride; RM ANOVA = repeated-measures ANOVA; mPFC = medial prefrontal cortex; OTR = oxytocin receptor.

PVT neurons are implicated in fear-related processes,^19,20^ but the role of OTR-expressing PVT neurons in fear extinction remains unclear. To investigate this, we chemogenetically suppressed neuronal activity during cue-conditioned fear-extinction learning (Fig. 3A). Fear memory acquisition did not differ between the experimental groups (Fig. 3B). Chemogenetic suppression had no effect on Day 1 (Fig. 3C) but significantly increased freezing levels on Day 2 and 1 week later (Fig. 3D and E), indicating impaired extinction. We further examined the effects of chemogenetically activating OTR-expressing PVT neurons during extinction learning in cue-conditioned settings. As before, fear memory acquisition did not differ between the groups (Fig. 3F). Activation significantly reduced freezing responses on Day 1 (Fig. 3G) but had no effect on Day 2 or 1 week later (Fig. 3H and I). These findings suggest that OTR-expressing PVT neurons play a crucial role in fear extinction, particularly under contextual and cue-conditioned settings. Chemogenetic suppression impaired extinction by increasing freezing responses, whereas activation enhanced fear extinction, primarily during the early stages of learning. In contrast, manipulating OTR-expressing mPFC neurons via CNO administration had no significant effect on fear extinction learning under cue-conditioned settings (Fig. 4A–I).

We further investigated the role of OTR-expressing PVT neurons in fear extinction by chemogenetically suppressing their activity during fear extinction learning in context-conditioned settings (Supplementary Fig. 9A). Fear memory acquisition did not differ between the experimental groups (Supplementary Fig. 9B). Suppression of PVT neuronal activity had no effect on fear extinction on Day 1 (Supplementary Fig. 9C) but significantly increased freezing responses on Day 2 and 1 week later (Supplementary Fig. 9D and E), indicating impaired extinction. Next, we examined whether chemogenetic activation of OTR-expressing PVT neurons during fear extinction learning could enhance extinction. Fear memory acquisition remained consistent across groups (Supplementary Fig. 9F). Chemogenetic activation significantly reduced freezing responses on Day 1 (Supplementary Fig. 8G) but had no effect on Day 2 or 1 week later (Supplementary Fig. 9H and I). In contrast, manipulation of OTR-expressing mPFC neurons via CNO administration did not significantly affect fear extinction learning under contextual conditions (Supplementary Fig. 10A–I).

### Oxytocin alters firing pattern-based subtypes of PVT neurons

**Figure 5:**
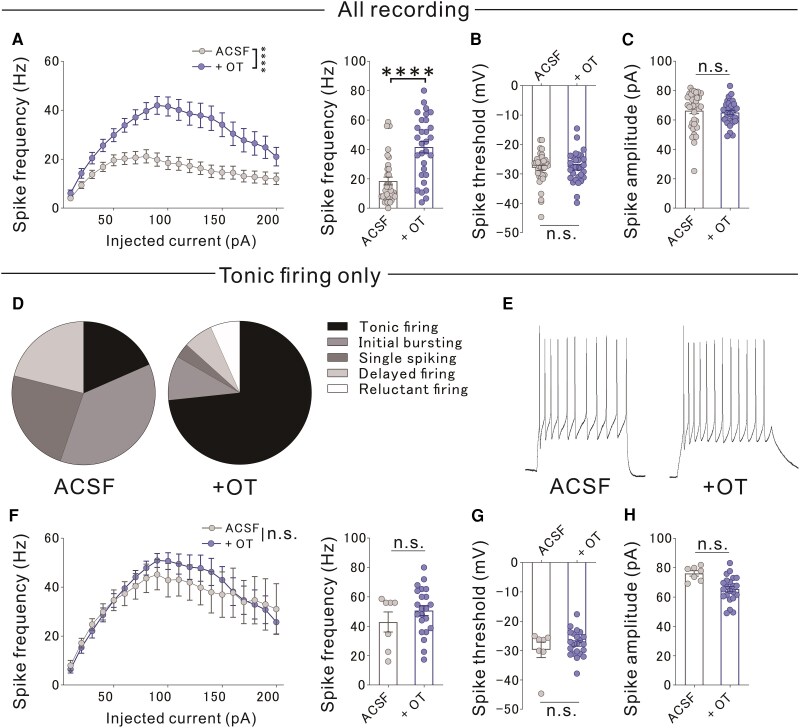
**Characteristics of OTR-expressing PVT neurons**. These electrophysiological recordings were conducted in mCherry-labelled OTR+ neurons without DREADD expression or CNO administration. (**A**) *Left*: Input-output curve in normal ACSF versus oxytocin-supplemented ACSF (+OT) showing increased excitability in OTR+ PVT neurons [two-way RM ANOVA, ACSF × Current-step *F*(19,1254) = 4.944, ****P < 0.0001; ACSF *F*(1,66) = 20.310, ****P < 0.0001; ACSF current-step *F*(2.398,158.2) = 22.180, ****P < 0.0001, normal ACSF: *n* = 38, +OT: *n* = 30]. *Right*: Spike frequency at the 100-pA was significantly higher in +OT condition (Mann–Whitney U-test, U = 227.5, ****P < 0.0001). (**B**) No significant difference in spike threshold of OTR-expressing PVT neurons (Mann–Whitney U-test, U = 541, *P =* 0.7242; normal ACSF: *n* = 38, +OT: *n* = 30). (**C**) No significant difference in spike amplitude in OTR-expressing PVT neurons (Mann–Whitney U-test, U = 483.5, *P =* 0.2888; normal ACSF: *n* = 38, +OT: *n* = 30). (**D**) Firing patterns of OTR+ PVT neurons (tonic, normal, single spiking, delayed, initial bursting) differed significantly (χ^2^ = 26.299, ****P < 0.0001). (**E**) Assessment of tonic firing intrinsic excitability of OTR-expressing neurons in normal ACSF and oxytocin (1 μM). Representative traces at 100 pA current injection from OTR-expressing PVT neurons (*n* = 30–38 cells per group). (**F**) *Left*: No significant input-output differences in a subset of OTR + PVT neurons [two-way RM ANOVA, ACSF component × Current-step interaction *F*(19,513) = 0.392, *P =* 0.9909; ACSF component effect *F*(1,27) = 0.156, *P =* 0.6916; current-step effect *F*(1.965,53.04) = 10.260, ***P = 0.0002, normal ACSF: *n* = 7, +OT: *n* = 22]. *Right*: No difference in spike frequency at 100 pA (Mann–Whitney U-test, U = 70, *P* = 0.7387; normal ACSF: *n* = 7, +OT: *n* = 22). (**G**) No difference in spike threshold (Mann–Whitney U-test, U = 70, *P* = 0.7387; normal ACSF: *n* = 7, +OT: *n* = 22). (**H**) OTR + PVT neurons in normal ACSF exhibited a significantly higher spike amplitude than +OT (two-tailed *t*-test, *t*_27_ = 3.014, **P = 0.0056; normal ACSF: *n* = 7, +OT: *n* = 22, +L368: *n* = 15). CNO = clozapine *N*-oxide dihydrochloride; RM ANOVA = repeated-measures analysis of variance; PVT = paraventricular thalamus; OT = oxytocin; OTR = oxytocin receptor; ACSF = artificial CSF; DREADD = designer receptors exclusively activated by designer drugs.

Oxytocin neurons project from the paraventricular hypothalamic nucleus to multiple brain regions, including the PVT.^38^ Although OTR agonist [(Thr4,Gly7)-Oxytocin] could offer greater receptor selectivity, we used oxytocin in slice preparations to assess the responsiveness of genetically defined OTR-expressing neurons, as a proxy for endogenous receptor activation. To investigate whether oxytocin induces dysfunction in OTR-expressing PVT neurons, we performed patch-clamp recordings in Oxtr-Cre mice treated with the OTR agonist. Treatment with oxytocin enhanced the intrinsic excitability of these neurons (Fig. 5A) and affected the balance of excitatory and inhibitory postsynaptic currents (Supplementary Fig. 11A–J); however, it did not alter the spike threshold or amplitude between the two groups (Fig. 5B and C). We classified PVT neurons into five subtypes based on their firing patterns^39^ (Supplementary Fig. 12). Oxytocin increased tonic firing compared with baseline conditions (Fig. 5D and E) but did not significantly affect spike frequency, threshold or amplitude within this tonic firing group (Fig. 5F–H). Thus, rather than uniformly increasing spike frequency, oxytocin shifted the firing distribution towards tonic firing, thereby enhancing overall PVT excitability.

### Salivary oxytocin levels correlate with sociability and fear in humans

Given the significant correlation between OTR-expressing PVT neurons and sociability/fear behaviours in mice, we examined the relationship between salivary oxytocin levels and brain microstructure in children with ASD. Salivary oxytocin concentrations were measured, and 3.0-Tesla MRI was performed to assess brain morphology. Neurite orientation dispersion and density imaging was used to quantify the neuronal microstructure, evaluating the angular variation in neurites (via the ODI) and axonal/dendritic densities (via the NDI).^40,41^  Supplementary Table 1 summarizes the demographic characteristics of participants in the ASD and TD groups.

ASD symptoms were assessed using the AQ-J.^33^ Age and sex did not differ significantly between the ASD and TD groups, nor did the NDI or ODI values in the thalamus or DLPFC. Given the presence of OTR-expressing neurons in these regions, we examined associations between salivary oxytocin levels and NDI/ODI values in the thalamus and DLPFC using multiple linear stepwise regression. Salivary oxytocin levels were significantly correlated with thalamic NDI across all participants (Supplementary Table 2). Notably, thalamic NDI—but not DLPFC NDI—was significantly associated with AQ-J total scores, as well as attention switching, communication subscales, and GHQ-30 measures of anxiety, dysthymia, suicidal ideation and depression (Table 1). However, ODI values in both regions showed no significant associations with AQ-J scores (Supplementary Table 3). In addition, Pearson’s correlation analysis was performed to further examine the relationship between salivary oxytocin levels and brain microstructure. As shown in Supplementary Tables 4–6, significant positive correlations were observed in the TD group between oxytocin levels and thalamic NDI (r = 0.829, *P* = 0.006) and ODI (r = 0.681, *P* = 0.043). No such correlations were found in the ASD group. These findings complement the multiple regression results and highlight the specificity of thalamic associations in ASD individuals.

**Table 1:** Multiple regression analysis of variables associated with the psychiatric symptoms in patients with ASD and TD individuals

Dependent variable and covariate	B	SE	β	*t*-value	*P*-value	R^2^	Adjusted R^2^	*P*-value
AQ-J total	–	–	–	–	–	0.275	0.191	**0**.**037**
NDI thalamus	141.427	71.439	0.383	1.980	0.058	–	–	–
NDI DLPFC	255.326	146.790	0.305	1.739	0.094	–	–	–
OT	−0.186	0.103	−0.337	−1.804	0.083	–	–	–
AQ-J social skill	–	–	–	–	–	0.164	0.068	0.191
AQ-J attention switching	–	–	–	–	–	0.314	0.235	**0**.**019**
NDI thalamus	38.607	16.079	0.451	2.401	**0**.**024**	–	–	–
NDI DLPFC	56.575	33.038	0.292	1.712	0.099	–	–	–
OT	−0.043	0.023	−0.339	−1.870	0.073	–	–	–
AQ-J local detail	–	–	–	–	–	0.089	−0.016	0.478
AQ-J communication	–	–	–	–	–	0.347	0.272	**0**.**010**
NDI thalamus	46.197	19.480	0.435	2.371	**0**.**025**	–	–	–
NDI DLPFC	32.336	40.028	0.135	0.808	0.427	–	–	–
OT	−0.096	0.028	−0.606	−3.418	**0**.**002**	–	–	–
AQ-J imagination	–	–	–	–	–	0.187	0.094	0.139
GHQ-30 general disease trends	–	–	–	–	–	0.125	0.024	0.315
GHQ-30 physical conditions	–	–	–	–	–	0.039	−0.072	0.788
GHQ-30 sleeping disorder	–	–	–	–	–	0.156	0.058	0.214
GHQ-30 social activity disorder	–	–	–	–	–	0.291	0.209	**0**.**028**
NDI thalamus	11.788	10.288	0.219	1.146	0.262	–	–	–
NDI DLPFC	53.378	21.139	0.438	2.525	**0**.**018**	–	–	–
OT	−0.002	0.015	−0.025	−0.138	0.891	–	–	–
GHQ-30 anxiety and dysthymia	–	–	–	–	–	0.291	0.209	**0**.**028**
NDI thalamus	34.110	13.920	0.468	2.450	**0**.**021**	–	–	–
NDI DLPFC	41.046	28.601	0.249	1.435	0.163	–	–	–
OT	−0.031	0.020	−0.282	−1.527	0.139	–	–	–
GHQ-30 suicidal ideation and depression	–	–	–	–	–	0.427	0.361	**0**.**002**
NDI thalamus	51.061	14.463	0.606	3.530	**0**.**002**	–	–	–
NDI DLPFC	31.641	29.718	0.166	1.065	0.297	–	–	–
OT	−0.068	0.021	−0.543	−3.273	**0**.**003**	–	–	–

Bold values indicate statistically significant differences. ASD = autism-spectrum disorder; TD = typically developing; SE = standard error; NDI = neurite density index; AQ-J = Japanese version of the Autism-Spectrum Quotient; OT = oxytocin; GHQ-30 = a 30-item General Health Questionnaire; DLPFC = dorsolateral prefrontal cortex.

These findings suggest that ASD symptoms may be more closely linked to thalamic microstructure than to that of the DLPFC in humans. Specifically, the association between salivary oxytocin levels and thalamic NDI suggests a potential role of the thalamus in modulating sociability and fear-related behaviours in ASD.

## Discussion

OTR-expressing PVT neurons modulate both social behaviour and fear-related responses in mice. Chemogenetic suppression of these neurons reduced sociability and impaired fear extinction, whereas activation enhanced fear extinction learning. This experimental approach was motivated by growing clinical evidence that individuals with ASD often exhibit post-traumatic stress disorder-like symptoms, including exaggerated threat responses and impaired fear regulation.^3^ Our findings suggest that OTR-expressing PVT neurons may contribute to the regulation of maladaptive fear responses associated with these comorbid features. Although the experimental approaches differed across species, salivary oxytocin levels in humans were modestly correlated with thalamic microstructure and ASD symptom severity. These findings suggest a possible role of the PVT in social and emotional behaviour, with potential relevance to psychiatric conditions. Moreover, the contrasting effects of OTR-related modulation observed in the PVT versus the mPFC highlight putative region-specific functions of oxytocin, positioning the PVT as a candidate hub for integrating social and emotional signals.

Bidirectional modulation of sociability and fear extinction learning in mice was observed following the chemogenetic manipulation of OTR-expressing PVT neurons, whereas the manipulation of OTR-expressing mPFC neurons had no effect on sociability. Oxytocin has been reported to modulate social behaviour via OTRs in various brain regions, including the nucleus accumbens and auditory cortex,^42,43^ and to facilitate social approach via the mPFC in oestrous females.^44^ However, evidence for similar effects in males remains limited. Although our results highlight the role of OTR-expressing PVT neurons in social behaviour, further research is needed to determine whether these effects are mediated by endogenous oxytocin. Our findings suggest that oxytocin may modulate sociability in males. Beyond social behaviours, oxytocin is critical for regulating anxiety and stress,^45^ exhibiting anxiolytic effects in both pharmacological and genetic studies. Although sex differences in oxytocin responses are well-documented in humans,^46^ the higher-order neural circuits underlying its anxiolytic effects—and their sex-specific features—remain poorly understood. The PVT emerges as a key hub in the oxytocin-associated regulation of sociability and anxiety-related behaviours, potentially linking social interactions with stress modulation, and suggesting an integration function across these behavioural domains. Clarifying the role of OTR-expressing PVT neurons in these processes, and identifying the conditions under which endogenous oxytocin is engaged, may offer valuable insights into the interaction between social behaviour and anxiety regulation.

Oxytocin exerts its excitatory effect on the neurons of various brain regions by activating OTRs, which are Gq-type G-protein coupled receptors.^47-49^ This activation triggers intracellular signalling cascades, leading to depolarization through transient receptor potential C channels and Na^+^/Ca^2+^ exchangers, along with the involvement of associated second messengers.^47,48^ Increased tonic firing in the PVT may influence social behaviour and facilitate fear extinction by modulating stress-related circuits, although a direct link between oxytocin-driven neuronal activity and these behavioural outcomes has yet to be established. Sustained post-stress PVT activity affects maladaptive behaviours, including those observed in acute stress disorder. Inhibition of these neurons alleviates symptoms, highlighting their role in stress-related affective dysfunction.^50^ The PVT plays a significant role in emotional processing, affecting arousal and valence,^51^ and is essential for fear extinction memory retrieval^19,20^ and social behaviour.^24^ Although our *ex vivo* electrophysiological data obviously indicate that oxytocin enhances excitability in PVT neurons, whether endogenous oxytocin is released during social or fear-related behavioural tasks *in vivo* remains unknown.

Li et al.^37^ demonstrated that the optogenetic activation of OTR-expressing interneurons in the mPFC enhanced social discrimination in female mice, whereas in our study, the chemogenetic activation of a broader population of OTR-expressing neurons in male mice had no effect on social preference. This discrepancy may reflect differences in sex, behavioural paradigms and neuronal subtypes, highlighting the need to consider the sex- and task-specific roles of mPFC microcircuits. Our immunohistochemical analysis further revealed that, unlike the PVT where OTR-expressing neurons were almost exclusively excitatory, the mPFC contained a substantial inhibitory subset (∼25%). This cellular heterogeneity may have contributed to the lack of behavioural effects by dampening or balancing the net impact of chemogenetic manipulation. Consistent with this, our chemogenetic manipulation of OTR-expressing mPFC neurons did not significantly affect sociability or fear extinction in male mice; this could be attributed to distinct circuit specializations^52^ and region-specific OTR dynamics.^53^ PVT activation also influences the excitation/inhibition balance in the mPFC, impacting mood stability and social behaviour.^54^ Increased PVT activity during stress can disrupt dorsomedial PFC function, a key region for fear memory processing.^55^ These findings indicate that the PVT and mPFC are functionally connected, whereas the behavioural effects of oxytocin are primarily mediated through PVT activity rather than through direct actions on OTR-expressing mPFC neurons. Our previous study established a causal link between juvenile social isolation, mPFC–PVT circuit disruption and persistent sociability deficits in C57BL/6J mice.^24^ In the present study, we extend this line of investigation by targeting OTR-expressing PVT neurons to characterize their role in modulating sociability and fear-related behaviours under non-stressed conditions. Although this approach does not model ASD, it provides a circuit-level framework that may inform future studies using ASD-relevant animal models.

In this study, we explored the relationship between oxytocin, brain structure and ASD symptoms using neurite orientation dispersion and density imaging. Significant associations were identified between salivary oxytocin levels and both NDI and ODI in the thalamus. Salivary oxytocin levels positively correlated with thalamic microstructure, highlighting a potential role of oxytocin in shaping neuronal morphology. Notably, thalamic NDI was specifically linked to ASD symptoms, particularly in attention switching and communication. In contrast, no significant correlation was observed between salivary oxytocin levels and NDI in the DLPFC. Although this human dataset did not assess brain activity or receptor-level function directly, the observed correlations suggest a possible link between peripheral oxytocin levels and thalamic structure. The findings suggest that oxytocin may influence thalamic microstructure, potentially contributing to the expression of ASD-related symptoms. Given the role of the thalamus in sensory processing and attention, it may represent a key target for future ASD research and therapeutic intervention. Genetic variations in the OTR gene have been associated with psychiatric disorders, including ASD and mood disorders.^56,57^

Studies examining plasma oxytocin levels in individuals with social anxiety disorder have yielded mixed results, with some suggesting improvement via adjunct oxytocin therapy.^58^ These findings imply that oxytocin may selectively enhance specific aspects of social functioning rather than offering universal benefit. Individuals with ASD often display broad social dysfunction, including reduced social motivation, social aloofness, diminished eye contact and difficulty maintaining social relationships.^59^ Intranasal oxytocin administration has shown promise in addressing these deficits, improving behaviours such as eye gaze, trust, affective speech recognition and performance on tasks like the ‘Reading the Mind in the Eyes Test’.^60,61^ These findings suggest that the effects of oxytocin on social anxiety and ASD are nuanced and context dependent, necessitating further investigation to clarify the underlying mechanisms. Moreover, group-specific correlation patterns provide additional insight. Although the TD participants exhibited strong positive correlations between oxytocin levels and thalamic microstructure, these associations were absent in the ASD group. This difference could suggest altered responsiveness to endogenous oxytocin or differences in receptor availability, although our study did not directly assess central oxytocin dynamics or receptor function. It may also reflect reduced thalamic sensitivity in ASD. Together with our regression results, these findings reinforce the thalamus as a key region mediating the relationship between peripheral oxytocin levels and social-emotional functioning. Elucidating the relationship between oxytocin and other neurotransmitter systems may offer valuable insights into the development of more targeted and effective treatments for social dysfunction in neurodevelopmental and psychiatric disorders. However, these results should be interpreted with caution, as the relationship between peripheral and central oxytocin concentrations remains controversial. A systematic review and meta-analysis reported no significant correlation between salivary or plasma oxytocin levels and cerebrospinal fluid oxytocin concentrations under basal conditions, suggesting that peripheral oxytocin may not reliably reflect central oxytocin activity.^62^ Although some studies in non-human primates have shown that intranasal oxytocin can attenuate stress responses, such as reduced adrenocorticotropic hormone release following administration,^63^ the extent to which such peripheral manipulation reflects endogenous oxytocin signalling in the brain remains unclear. Our findings are therefore exploratory in nature and should be confirmed using direct measures of central oxytocin dynamics or receptor engagement.

OTR-expressing PVT neurons regulate social behaviour and fear, highlighting their critical role in emotional processing. Thus, oxytocin-related PVT activity may represent a promising therapeutic target for ASD and anxiety disorders, both of which involve impaired social cognition and exaggerated fear responses. The PVT appears to function as a nexus mediating the reciprocal relationship between social and fear-related behavioural and mental processes. However, our chemogenetic approach modulates the activity of OTR-expressing neurons without directly implicating the involvement of endogenous oxytocin in these behaviours. Further research is needed to elucidate the specific neural circuits and molecular mechanisms underlying the oxytocin-related activity in the PVT that modulates these behaviours. Translational studies are also essential to determine whether pharmacological modulation of this system could provide therapeutic benefits in humans.

Although triadic and tetradic interaction metrics could yield deeper insights into higher-order group dynamics, our use of unfamiliar cage-mates in the study design reduces the relevance of such analyses. Instead, we employed the switching ratio as a relevant and interpretable measure of dyadic partner diversity. Given that individuals with ASD often exhibit pronounced behavioural alterations in novel contexts,^3^ we focused on the first 60 min of social exposure to capture potential abnormalities during this critical period of adaptation. This approach enhances the translational relevance of our findings to ASD-related social deficits.

This study has several limitations. First, most experiments were conducted exclusively in male mice. Given the well-documented sex differences in oxytocin-related effects, the findings may not be generalizable to females. Although both human and animal cohorts were predominantly male, they differed in developmental stage: the human participants were early adolescents (∼14 years old), whereas the mice were young adults (10–15 weeks). Considering the age-dependent plasticity of oxytocin-related effects and social behaviour, this developmental mismatch should be considered when interpreting translational relevance. Second, the human data are correlational and do not establish causality, and the sample size was relatively small. Larger and more diverse cohorts will be necessary to validate associations between oxytocin, brain structure and ASD symptoms. Third, the potential compensatory mechanisms and long-term consequences of manipulating OTR-expressing neurons were not explored. Chronic interventions may yield different outcomes from acute manipulations. Fourth, only salivary oxytocin levels were measured in humans. Although cerebrospinal fluid or direct brain oxytocin measurements would more accurately represent central oxytocin availability, they were not assessed in this study. Although we focused on resting-state oxytocin and magnetic resonance imaging, previous research has shown that baseline oxytocin levels are associated with social cognition and brain structure,^64^ and that altered baseline oxytocin levels are observed in ASD.^60^ Future studies incorporating socially interactive paradigms during oxytocin sampling may provide further insight into the state-dependent dynamics of the oxytocin system in ASD.^60^ Fifth, the study primarily focused on the PVT and mPFC, despite the widespread influence of oxytocin across multiple brain regions. A more comprehensive mapping of oxytocin-related circuitry is warranted. Sixth, potential interactions with other neurotransmitter systems were not examined, even though oxytocin likely interacts with multiple neuromodulatory processes. Seventh, salivary oxytocin may not directly reflect central oxytocin levels, as it is influenced by peripheral factors and does not cross the blood–brain barrier. However, recent studies have reported altered salivary oxytocin levels in individuals with ASD compared with TD controls,^65^ supporting its potential utility as a peripheral biomarker for social and emotional function. Eighth, our DREADD-based manipulation does not replicate endogenous oxytocin activity, but modulates the activity of all OTR-expressing neurons, both excitatory and inhibitory, due to the use of a synapsin promoter. Thus, behavioural effects likely reflect the aggregate function of this mixed population, rather than specific OTR engagement.

## Conclusion

OTR-expressing PVT neurons regulate social behaviour and fear responses in mice, as shown by reduced sociability and impaired extinction learning in separate tasks following chemogenetic suppression. In contrast, chemogenetic activation enhanced early extinction learning but had no detectable effect on sociability. Oxytocin administration increased tonic firing of PVT neurons, whereas manipulation of OTR-expressing mPFC neurons had no effect on sociability. In humans, salivary oxytocin levels correlated with thalamic microstructure and ASD symptoms, highlighting the potential role of oxytocin-related PVT function in adaptive social and emotional processing, with implications for psychiatric disorders.

This study advances our understanding of the role of oxytocin in regulating social behaviour and emotional responses. The PVT emerges as a critical hub integrating social and fear-related behaviours, offering insights into both fundamental neuroscience and the development of clinical interventions for psychiatric conditions.

## Supplementary Material

awaf421_Supplementary_Data

## Data Availability

The data that support the findings of this study are available from the corresponding author, upon reasonable request.
