# Supplementary material for: Oxytocin receptor neurons in the paraventricular thalamus as a nexus for social behaviour and fear
Source: Brain. 2026 Mar 20;149(6):2151–65. doi: 10.1093/brain/awaf421 (PMC13233147; doi:10.1093/brain/awaf421)
Supplement: awaf421_Supplementary_Data [file awaf421_supplementary_data.pdf]

# Supplementary Materials

## Oxytocin receptor neurons in the paraventricular thalamus as a nexus for social behaviour and fear

Kazuhiko Yamamuro[1,2,†], Minobu Ikehara[2,†], Yuki Noriyama[2], Mamiko Okuda[2], Kazuki Okumura[2], Kiwamu Matsuoka[2], Natsuko Kashida[2], Rio Ishida[2,3,4,5], Tsutomu Takeda[2,3,4,5], Michihiro Toritsuka[2,3,4,5], Tomoko Ochi[6], Toshiteru Miyasaka[6], Yumi Tai[6], Kouko Tatsumi[7], Tsuyoshi Hattori[7], Toshihiro Tanaka[6], Yasuhiko Saito[8], Nakao Iwata[3], Manabu Makinodan[2,3,4,5]

[†]**These authors contributed equally to this work.**

1 Center for Health Control, Nara Medical University; Kashihara, Nara 634-8521, Japan

2 Department of Psychiatry, Nara Medical University; Kashihara, Nara 634-8521, Japan

3 Department of Psychiatry, Fujita Health University; Toyoake, Aichi 470-1192, Japan

4 Division of Transformative Psychiatry and Synergistic Research, International Center for Brain Science, Fujita Health University; Toyoake, Aichi 470-1192, Japan

5 Department of Neuropsychiatry, Kumamoto University, Kumamoto, Kumamoto 860-8555, Japan

6 Department of Diagnostic and Interventional Radiology, Nara Medical University; Kashihara, Nara 634-8521, Japan

7 Department of Anatomy and Neuroscience, Nara Medical University; Kashihara, Nara 634-8521, Japan

8 Department of Neurophysiology, Nara Medical University; Kashihara, Nara 634-8521, Japan

**Corresponding authors**

*Kazuhiko Yamamuro

Center for Health Control, Nara Medical University, 840 Shijyo-Cho, Kashihara, Nara 634-8521, Japan

E-mail: muro@naramed-u.ac.jp

*Manabu Makinodan

Department of Neuropsychiatry, Kumamoto University, Kumamoto, Kumamoto 860-8555, Japan

E-mail: manabu.makinodan@fujita-hu.ac.jp

**This PDF file includes:**

Materials and Methods

Supplementary Figures 1 to 12

Supplementary Tables 1 to 6

# Materials and Methods

## Stereotaxic surgery

Mice were anesthetised with isoflurane delivered via inhalation and secured in a stereotaxic apparatus (Narishige, Tokyo, Japan) to ensure precise head fixation. For chemogenetic studies, bilateral viral injections were administered in 6–7-week-old mice using the following stereotaxic coordinates: For paraventricular thalamus (PVT) injections, two sites per hemisphere were targeted to ensure broad coverage: AP = −1.5 mm and −1.8 mm; ML = 0 mm; DV = −2.8 mm. A total of four injections (two per hemisphere) were administered per animal. For medial prefrontal cortex (mPFC) injections, three sites per hemisphere were used: AP = +2.3 mm, +2.0 mm, and +1.7 mm; ML = ±0.4 mm; DV = −1.3 mm. A total of six injections (three per hemisphere) were administered per animal. Each injection contained one of the following AAVs: AAV8-hSyn-DIO-hM4Di-mCherry (iDREADD) (44362-AAV8: Addgene), AAV8-hSyn-DIO-hM3Dq-mCherry (eDREADD) (44361-AAV8: Addgene), or AAV8-hSyn-DIO-mCherry (mCherry-Cre) (50459-AAV8: Addgene). A total volume of 400 nL per injection was infused at a rate of 180 nL/min using a microinjector and glass pipettes.

## Validation of cell-type specificity

To ensure cell-type specificity, we used $\mathit{Oxtr}^{tm1(cre/GFP)Rpa}$/J (Oxtr-Cre) mice in combination with Cre-dependent AAV vectors (AAV8-hSyn-DIO-hM4Di-mCherry or AAV8-hSyn-DIO-hM3Dq-mCherry). Expression of mCherry was observed in the PVT or mPFC, depending on the injection site—both regions where oxytocin receptor (OTR)-expressing neurons are known to reside. Cell-type specificity in this system is conferred by *Oxtr* promoter-driven expression of Cre recombinase. The reliability of this targeting strategy has been validated by Newmaster et al.,[1] who showed that

Cre expression in *Oxtr*$^{tm1(cre/GFP)Rpa}$/J mice faithfully recapitulates endogenous *Oxtr* mRNA expression throughout the mouse brain using whole-brain cellular-resolution mapping.[1] All experimental animals underwent histological validation for mCherry expression, which was consistently localised to the targeted brain regions (PVT or mPFC). Reconstructions of the injection sites across animals are provided in Supplementary Figs. 1 and 2.

Histological validation of mCherry expression was performed in a subset of the animals that underwent behavioural testing, to confirm the anatomical specificity of viral targeting. Mice were anesthetised with isoflurane and transcardially perfused with cold 0.1 M phosphate buffer, followed by 4% paraformaldehyde in 0.1 M phosphate buffer. Their brains were post-fixed overnight at 4 °C in the same fixative and subsequently cryoprotected in 30% sucrose. Frozen brains were coronally sectioned at 35 μm using a cryostat (HM525NX: Thermo Fisher Scientific). Free-floating sections were washed three times in phosphate-buffered saline containing 0.3% Triton X-100 (PBST) and then blocked for 30 min in PBST containing 5% normal donkey serum (AB_2337254: Jackson ImmunoResearch Laboratories). Double immunofluorescence staining was performed by simultaneous incubation with the following primary antibodies: guinea pig anti-vGluT1 (136304: Synaptic Systems; 1:500), guinea pig anti-vGluT2 (135404: Synaptic Systems; 1:500), rabbit anti-Cux1 (sc-22200: Santa Cruz Biotechnology; 1:200), rat anti-Ctip2 (ab18465: Abcam; 1:1000), mouse anti-GAD65 (198111: Synaptic Systems; 1:200), and mouse anti-GAD67 (ab26116: Abcam; 1:200). After washing in PBST, sections were incubated with the appropriate secondary antibodies: Alexa Fluor 488 donkey anti-guinea pig IgG (H+L) (706-545-148: Jackson ImmunoResearch Laboratories; 1:1000), Alexa Fluor 488 donkey anti-rabbit IgG (H+L) (711-545-152: Jackson ImmunoResearch Laboratories; 1:1000), Alexa Fluor 488 donkey anti-rat IgG (H+L) (712-545-153: Jackson ImmunoResearch Laboratories; 1:1000), and Alexa Fluor 647 donkey anti-

mouse IgG (H+L) (A-31571: Thermo Fisher Scientific; 1:1000). Blue-fluorescent Nissl staining was performed simultaneously using NeuroTrace 435/455 (N21479: Thermo Fisher Scientific; 1:500) as a counterstain. Sections were mounted with VECTASHIELD (H-1700: Vector Laboratories) and stored for imaging. Fluorescent images were acquired using an all-in-one fluorescence microscope (BZ-X800: Keyence).

## Behavioural testing

For iDREADD and eDREADD manipulations, clozapine N-oxide dihydrochloride (CNO) (Tocris Bioscience, Bristol, London) was dissolved in saline and administered via intraperitoneal injection, 30 min before behavioural testing, at a dose of 5 mg/kg body weight (iDREADD experiments) or 1 mg/kg body weight (eDREADD experiments). These doses—5 mg/kg for hM4Di (iDREADD) and 1 mg/kg for hM3Dq (eDREADD)—were selected based on previous reports demonstrating their effectiveness and selectivity.[2–4] All behavioural experiments (three-chamber, elevated plus maze, open field, the augmented reality-based long-term animal behaviour observing system [AR-LABO], and fear conditioning) were conducted using independent cohorts for eDREADD and iDREADD manipulations. Within each cohort, CNO and saline treatments were administered to separate animals in an alternating (interleaved) order. No animals were shared across cohorts or behavioural tasks. The 30-min pre-treatment timing was selected based on previous studies showing that DREADD-mediated effects typically peak around 30–45 min and persist for at least 90 min.[4–7] Therefore, the 60-min AR-LABO session was conducted entirely within the expected effective window. Mice received both CNO and saline treatments, with a minimum of 1-week of washout period between experiments. Animals displaying significant motor impairments due to

intraperitoneal procedures were excluded from the testing. All behavioural experiments were initiated when the animals were 9–10 weeks old.

In addition to the primary experimental groups targeting the PVT, we also performed parallel experiments targeting the mPFC, a brain region independently implicated in social behaviour and fear processing. These experiments were not intended as control groups, but rather to assess whether chemogenetic manipulation of OTR-expressing neurons in the mPFC would produce behavioural effects similar to those observed with PVT manipulation. As no significant effects were observed in the mPFC group, these findings serve to highlight the regional specificity of the PVT effects.

## Three-chamber test

The apparatus consisted of a three-compartment rectangular structure with grey acrylic walls and a base (61 cm width × 40 cm depth × 30 cm height; O'Hara & Co., Ltd.), as described in our previous studies.[3,4,8] Mouse behaviour was recorded and analysed using the ANY-maze system, version 7.0 (Stoelting Co., Wood Dale.). Social interaction time ("mouse interaction time" or "object interaction time") was defined as the total time spent in the interaction zone—a circular area extending 3 cm around the corral containing either the mouse or the object.

## Elevated plus maze

The elevated plus maze is a widely used assay for assessing anxiety-related and exploratory behaviour in rodents.[3,4] It involves comparing the time spent in the enclosed arms of a plus-shaped maze with four equal-sized arms elevated above the ground. Mice were placed in the central square

and allowed to explore freely for 8 min. Behaviour was recorded and analysed using the ANY-maze system.

## Open field

To assess anxiety-related and locomotor behaviours, mice were placed in a square acrylic arena (43 × 43 × 33 cm; O'Hara & Co., Ltd.). Behaviour was recorded and analysed using the ANY-maze system, which measured exploratory behaviour and locomotor activity over a 30-min period.[3,4] Anxiety levels were evaluated based on the time spent in the centre versus the periphery, whereas locomotor activity was measured by total distance travelled.

## Fear conditioning

Fear-conditioning experiments were conducted as previously described.[9] Auditory fear conditioning was performed in a clear, square-shaped arena (170 × 100 × 100 mm) with a metal grid shock floor for delivering scrambled footshocks (unconditioned stimulus; 0.3 mA, 1 s; O'Hara & Co., Ltd.). The arena was placed in an acoustic isolation box (background noise: 50 dB, illumination: 200 lx), with conditioning stimuli (white noise, 65 dB, 10 s) delivered through a speaker positioned on the right side of the chamber. On the training day, each mouse was placed in the arena and allowed to explore freely for 1 min. The conditioning stimuli was then presented for 10 s, with a 0.3 mA footshock applied during the final second. The conditioning stimuli–unconditioned stimulus pairing, which was repeated twice at 20 s intervals, was delivered automatically by the tone generator and shock controller. Mice remained in the arena for 3 min before being returned to their home cages. Twenty-four hours later, a contextual fear-conditioning test was conducted in the same arena without conditioning stimuli or unconditioned stimulus

presentation. Mice were placed in the chamber for 6 min. Another 24 h later, a cued test was performed in a solid, grey, square-shaped arena within an acoustic isolation box (background noise: 55 dB, illumination: 50 lx). Fear extinction sessions lasted 6 min in total, consisting of 3 min of free exploration, followed by 1 min of tone (conditioning stimuli) presentation, and 2 min of free exploration post-conditioning stimuli.

Behavioural data were automatically collected and analysed using Time FZ1 (O'Hara & Co., Ltd.). Freezing behaviour, defined as motionless periods lasting ≥2 s, was measured as the percentage of the total session time.

.

## Augmented reality-based long-term animal behaviour observing system

We applied our assay system for precise tracking and analysis of social behaviour in animals within a novel environment. This system determines the position of each mouse under social housing conditions. At least one week before behavioural testing, each mouse was tagged with an identification (ID) marker (printed with ArUco markers) on the back, attached via an elastic string under anaesthesia (chloral hydrate 400 mg/kg, intraperitoneal administration). To prevent cage-mates from biting the ID tags, they were coated with quinine. This tagging method has been validated in previous AR-LABO studies and does not affect sociability.[8] In the behavioural experiment, one subject mouse and three age- and sex-matched C57BL/6J mice (that had never been co-housed with the subject mouse) were placed in a cage (276 × 445 × 204 mm; CL-0128: CLEA Japan Inc.) and allowed to interact freely for 1 h. Each test mouse was introduced into a novel cage with three age- and weight-matched C57BL/6J males that had been co-housed with the test mouse for at least two weeks prior to testing. The behaviour of all four mice was monitored at 20 frames/s using an infrared camera under infrared illumination. The video data were analysed

offline using an in-house tracking system[2] (now available at O’Hara & Co., Ltd.). The central position of each mouse's ID tag was detected in each frame as XY coordinates within the cage. The time-series coordinate data were exported as comma-separated values (.csv) files. Subsequent data processing was carried out using the same algorithm as previously described[8], employing Python 3.10.8 and in-house programmes. Missing coordinates were linearly interpolated between detected frames.

Locomotor activity was quantified as the total distance moved (in meters) by the ID tag of each mouse within a specified time period. The total distance travelled was calculated as the sum of the frame-to-frame displacements, with each displacement defined as the Euclidean distance between the positions of the same mouse in consecutive video frames throughout the analysis period. To analyse social interactions, a contact event was defined when the ID tags of two mice were within 20 mm of each other. Contact initiation was classified based on the distances moved by each mouse in the previous second (20 frames): the mouse moving the longer distance was labelled as the approaching mouse and the other mouse (moving the shorter distance) as the receiving mouse. Contact initiated by the approaching mouse was categorised as active contact, whereas contact experienced by the receiving mouse was categorised as passive contact. Subsequently, contact frequency and duration were calculated for each mouse in both active and passive roles. In addition, to assess whether a mouse was biased toward a specific cage-mate an entropy measure (**$H_i$**) was calculated based on the probability ($p_{ij}$) of contacting different mice using the following formula: $H_i = -\Sigma_j \{p_{ij} \log_2 (p_{ij})\}$, where $Hi$ represents the entropy of social interactions for mouse $I$; $p_{ij}$ is the probability that mouse $i$ interacts with mouse $j$ (either in terms of number of interactions or total interaction duration); and the summation $\sum_j$ runs over all potential interaction partners $j$ (i.e., the three cage-mates of mouse $i$).

## Electrophysiology

Brain slices containing the PVT were prepared from 61-day-old mice. Animals were deeply anaesthetised with isoflurane and decapitated, after which the brain was quickly removed and immersed in an ice-cold (approximately 4 °C) sucrose-based solution bubbled with a 95% $O_2$/5% $CO_2$ gas mixture. The solution contained (in mM): 210.3 sucrose, 2.5 KCl, 26.2 $NaHCO_3$, 1.0 $NaH_2PO_4$, 0.5 $CaCl_2$, 4.0 $MgSO_4$, and 11.0 D-glucose. The frontal cortex was sectioned into 300 µm-thick coronal slices using a vibrating tissue slicer (Vibratome 1000 Plus 102, Pelco International, Redding). Slices were incubated for at least 60 min in a chamber filled with standard artificial cerebrospinal fluid (ACSF) (in mM: 119.0 NaCl, 2.5 KCl, 26.2 $NaHCO_3$, 1.0 $NaH_2PO_4$, 2.0 $CaCl_2$, 2.0 $MgCl_2$, and 11.0 D-glucose) at 32 °C , continuously bubbled with the same gas mixture, and then maintained in ACSF at 25 °C. Oxytocin (Sigma-Aldrich) was dissolved in ACSF at a final concentration of 1 µM and bath-applied during recordings. Measurements were obtained after at least 10 min of perfusion with oxytocin-containing ACSF. Following incubation, each slice was transferred to a recording chamber and superfused with ACSF (32 °C) at a flow rate of 2 mL/min, continuously saturated with the gas mixture.

OTR-expressing neurons were identified by mCherry fluorescence, driven by Cre-dependent AAV8-hSyn-DIO-mCherry (50459-AAV8: Addgene) injected into *Oxtr*-Cre mice. Neurons exhibiting mCherry signals were visually selected under epifluorescence and targeted for whole-cell patch-clamp recordings. Recordings were conducted in mCherry-labelled OTR-expressing neurons to evaluate the direct effects of oxytocin application. Electrophysiological recordings were performed only in the PVT. As our behavioural results showed no significant effect of OTR-expressing neuron manipulation in mPFC, electrophysiological experiments were focused

exclusively on the PVT, where the behavioural phenotypes were robust and reproducible. Venus-positive neurons were identified by fluorescence and patched under visual guidance using infrared differential interference contrast optics under an upright microscope (BX51WI: Olympus). Fluorescence images were obtained using an X-Cite XYLIS LED light source (model XT720S: Excelitas Technologies), a U-FMCHE fluorescence filter cube (customised for an Olympus BX51 microscope: Evident Scientific), and a DP23M digital microscope camera (Evident Scientific).

Cells were current- and voltage-clamped in a conventional whole-cell configuration using a Multiclamp 700A amplifier (Axon Instruments, Molecular Devices). Patch pipettes were made from borosilicate glass and had a resistance of 3–5 MΩ when filled with the intracellular solution. For current-clamp recordings, pipettes were filled with a low-chloride intracellular solution containing (in mM): 127.5 K-methanesulfonate, 5.0 KCl, 2.0 $MgCl_2$, 2.0 Mg-ATP, 0.3 Na-GTP, 0.6 ethylene glycol tetraacetic acid (EGTA), and 10.0 4-(2-hydroxyethyl)-1-piperazineethanesulfonic acid (HEPES); pH adjusted to 7.25 with KOH. For voltage-clamp recordings, an intracellular solution containing (in mM): 130.0 Cs-methanesulfonate, 8.0 NaCl, 4.0 Mg-ATP, 0.4 Na-GTP, 0.5 EGTA, 10.0 HEPES, 1.0 QX-314, and 10.0 Na-phosphocreatine was used; pH adjusted to 7.25 with CsOH. All membrane potentials were corrected for the liquid junction potential (10 mV for Cs-based solutions), measured as previously described.[4] Data acquisition and stimulation were controlled using Signal 4 software with a Power 1401 interface (Cambridge Electronic Design).

## Current-clamp recording

During current-clamp recordings, series resistance was monitored and compensated using a bridge circuit, and pipette capacitance was corrected. Voltage signals were low-pass filtered at 10 kHz

and digitised at 20 kHz. The baseline membrane potential was maintained near −70 mV via current injection. Action potential (AP) analysis was performed using Signal 4 software (version 4, Cambridge Electronic Design). The AP threshold was defined as the membrane potential at which the first derivative of the voltage trace (dV/dt) exceeded 10 mV/ms during a depolarising current step. The AP amplitude was measured as the voltage difference between the threshold and the peak of the first spike evoked by a 100-pA current injection.

To assess action potentials and subthreshold membrane properties, we recorded membrane potential responses to hyperpolarising and depolarising current pulses (500 ms duration). Depolarising pulses ranging 10–200 pA were applied in 10 pA increments.

## Voltage-clamp recording

During voltage-clamp recordings, pipette capacitance was compensated, while series resistance was continuously monitored but not compensated. Only recordings with a stable series resistance ≤20 MΩ were included in the analyses. Current signals were low-pass filtered at 800 Hz and digitised at a sampling frequency of 10 kHz. Spontaneous excitatory postsynaptic currents (EPSCs) and spontaneous inhibitory postsynaptic currents (IPSCs) were recorded in standard ACSF. EPSCs were recorded at a holding potential of −70 mV to isolate excitatory currents, and IPSCs at 0 mV to isolate inhibitory currents. Spontaneous EPSCs and IPSCs were analysed using Mini Analysis software (Synaptosoft Inc.). Events were detected using an amplitude threshold of 5 pA. A 10-min stable segment of continuous recording was selected for analysis.

## Human saliva, behavioural assessments, and neuroimaging data analysis

### Japanese version of the Autism-Spectrum Quotient

The Japanese version of the Autism-Spectrum Quotient (AQ-J) is a 50-item self-report questionnaire evaluating autistic traits across five core domains: social skills, attention switching, attention to detail, communication, and imagination. Each item is rated on a four-point Likert scale, with higher scores showing greater autistic traits. Example items include "I find it difficult to make new friends" (social skills), "I frequently get so absorbed in one thing that I lose sight of other things" (attention switching), and "I find it difficult to read between the lines in conversations" (communication). The AQ-J has been validated for use in Japanese populations and shows good internal consistency (Cronbach's $\alpha = 0.77–0.86$).[10]

### 30-Item General Health Questionnaire

Psychiatric symptoms were assessed using the 30-item General Health Questionnaire (GHQ-30),[11] a widely used self-report screening instrument for general psychological distress. Although originally developed for adults, the GHQ-30 has also been validated in adolescents aged 12 years and older,[12] indicating adequate suitability for younger populations in community and clinical settings. Based on this, the GHQ-30 was applied across our entire sample, including participants under 16 years of age.

### Salivary sample collection

Saliva samples were collected according to standardised protocols recommended by the Vanderbilt Hormone Assay & Analytical Services Core and Salimetrics SalivaLab (Carlsbad, CA, USA). The passive drool method, as endorsed by Salimetrics, was employed using Salivette collection tubes (51.1534.500: Sarstedt) to obtain 400 μL of clean saliva, ensuring sample integrity and compatibility with a range of analytes. Participants were instructed to refrain from brushing their teeth for at least 45 min prior to sample collection and to avoid dental procedures for 24 h beforehand. Samples were stored at −80 °C and subsequently shipped on dry ice to Salimetrics SalivaLab for analysis.

## Measurement of salivary oxytocin level

Saliva samples were analysed in triplicate at Salimetrics SalivaLab to quantify oxytocin concentrations using a validated electrochemiluminescence assay developed by Salimetrics. The average coefficient of variation for all samples tested was <20%–30%, meeting or exceeding the National Institutes of Health guidelines for Enhancing Reproducibility through Rigor and Transparency. Each assay was performed using 25 μL of saliva, with a lower sensitivity limit of 8 pg/mL and a dynamic range of 8–1000 pg/mL.

## Magnetic resonance imaging

Magnetic resonance imaging (MRI) scans were acquired using a 3.0-Tesla clinical scanner (Magnetom Skyra; Siemens, Erlangen, Germany) equipped with a 32-channel phased-array brain coil. High-resolution, three-dimensional T1-weighted anatomical images were collected using a magnetisation-prepared rapid gradient-echo sequence (repetition time [TR] = 2500 ms; echo time

[TE] = 2.18 ms; inversion time [TI] = 1000 ms; field of view [FOV] = 256 mm; flip angle = 8°; acquisition matrix = 320 × 300; sagittal slice thickness = 0.8 mm).

Neurite orientation dispersion and density imaging (NODDI) parameters were obtained using a two-shell diffusion MRI protocol. Diffusion-weighted images were acquired with an echo-planar imaging sequence (TR = 3600 ms; TE = 90 ms; b-values = 0, 700, 2000 s/mm$^2$; acquisition matrix = 120 × 120; flip angle = 90°). Motion probing gradients were applied in 67 anterior–posterior and 68 posterior–anterior directions.

## MRI data analysis

Diffusion-weighted images were processed using the Functional Magnetic Resonance Imaging tool of the Brain (FMRIB) Software Library (FSL) 6.0.6.2 (FMRIB Centre, Department of Clinical Neurology, University of Oxford, Oxford, UK; http://www.fmrib.ox.ac.uk/fsl/).[13] Susceptibility-induced off-resonance fields were estimated using the FSL *topup* tool,[14] which corrects distortions based on image pairs with reversed phase-encoding directions. Eddy current distortion and subject motion were subsequently corrected using FSL's *eddy* tool.[15] The NODDI model was fitted using the NODDI toolbox version 1.01 (https://www.nitrc.org/projects/noddi_toolbox/) running on MATLAB 2021a (MathWorks, Natick, MA, USA), generating orientation dispersion index (ODI) and neurite density index (NDI) value maps.

Surface-based cortical reconstruction and volumetric subcortical segmentation of T1-weighted images were performed using *FreeSurfer* software version 7.3.2 (https://surfer.nmr.mgh.harvard.edu/). Thalamic regions of interest were anatomically defined.[16] Based on a prior study,[17] the dorsolateral prefrontal cortex (DLPFC) region of interest was constructed by combining the middle frontal gyrus and the inferior frontal sulcus parcels from the

*aparc.a2009s* atlas.[18,19] Each participant's ODI and NDI maps were co-registered to a their three-dimensional T1-weighted image, and regional ODI/NDI values were calculated by averaging the values across all voxels within the defined regions of interest.

## References

1. Newmaster KT, Nolan ZT, Chon U, et al. Quantitative cellular-resolution map of the oxytocin receptor in postnatally developing mouse brains. *Nat Commun*. 2020;11(1):1885. doi:10.1038/s41467-020-15659-1.

2. Tiwari P, Kapri D, Pradhan A, Balakrishnan A, Chaudhari PR, Vaidya VA. Chronic hM4Di-DREADD-Mediated chemogenetic inhibition of forebrain excitatory neurons in postnatal or juvenile life does not alter adult mood-related behavior. *eNeuro*. 2022;9(1):ENEURO.0381-21.2021. doi:10.1523/ENEURO.0381-21.2021.

3. Bicks LK, Yamamuro K, Flanigan ME, et al. Prefrontal parvalbumin interneurons require juvenile social experience to establish adult social behavior. *Nat Commun*. 2020;11(1):1003.

4. Yamamuro K, Bicks LK, Leventhal MB, et al. A prefrontal–paraventricular thalamus circuit requires juvenile social experience to regulate adult sociability in mice. *Nat Neurosci*. 2020;23(10):1240-1252.

5. Roth BL. DREADDs for neuroscientists. *Neuron*. 2016;89(4):683-694. doi:10.1016/j.neuron.2016.01.040.

6. Manvich DF, Webster KA, Foster SL, et al. The DREADD agonist clozapine N-oxide (CNO) is reverse-metabolized to clozapine and produces clozapine-like interoceptive stimulus effects in rats and mice. *Sci Rep*. 2018;8(1):3840. doi:10.1038/s41598-018-22116-z.

7. Alexander GM, Rogan SC, Abbas AI, et al. Remote control of neuronal activity in transgenic mice expressing evolved G protein-coupled receptors. *Neuron*. 2009;63(1):27-39. doi:10.1016/j.neuron.2009.06.014.

8. Komori T, Okamura K, Ikehara M, et al. Brain-derived neurotrophic factor from microglia regulates neuronal development in the medial prefrontal cortex and its associated social behavior. *Mol Psychiatry*. 2024;29(5):1338-1349.

9. Shoji H, Takao K, Hattori S, Miyakawa T. Contextual and cued fear conditioning test using a video analyzing system in mice. *J Vis Exp*. 2014;(85):50871. doi:10.3791/50871.

10. Kurita H, Koyama T, Osada H. Autism-Spectrum Quotient–Japanese version and its short forms for screening normally intelligent persons with pervasive developmental disorders. *Psychiatry Clin Neurosci*. 2005;59(4):490-496. doi:10.1111/j.1440-1819.2005.01403.x.

11. Goldberg DP, Hillier VF. A scaled version of the General Health Questionnaire. *Psychol Med*. 1979;9(1):139-145. doi:10.1017/s0033291700021644.

12. Tait RJ, Hulse GK, Robertson SI. A review of the validity of the General Health Questionnaire in adolescent populations. *Aust N Z J Psychiatry*. 2002;36(4):550-557. doi:10.1046/j.1440-1614.2002.01028.x.

13. Smith SM, Jenkinson M, Woolrich MW, et al. Advances in functional and structural MR image analysis and implementation as FSL. *Neuroimage*. 2004;23(suppl 1):S208-S219. doi:10.1016/j.neuroimage.2004.07.051.

14. Andersson JLR, Skare S, Ashburner J. How to correct susceptibility distortions in spin-echo-planar images: application to diffusion tensor imaging. *Neuroimage*. 2003;20(2):870-888. doi:10.1016/S1053-8119(03)00336-7.

15. Andersson JLR, Sotiropoulos SN. An integrated approach to correction for off-resonance effects and subject movement in diffusion MR imaging. *Neuroimage*. 2016;125:1063-1078.

16. Fischl B, Salat DH, Busa E, et al. Whole brain segmentation: automated labeling of neuroanatomical structures in the human brain. *Neuron*. 2002;33(3):341-355. doi:10.1016/s0896-6273(02)00569-x.

17. Cosme D, Ludwig RM, Berkman ET. Comparing two neurocognitive models of self-control during dietary decisions. *Soc Cogn Affect Neurosci*. 2019;14(9):957-966. doi:10.1093/scan/nsz068.

18. Destrieux C, Fischl B, Dale A, Halgren E. Automatic parcellation of human cortical gyri and sulci using standard anatomical nomenclature. *Neuroimage*. 2010;53(1):1-15. doi:10.1016/j.neuroimage.2010.06.010.

19. Fischl B, van der Kouwe A, Destrieux C, et al. Automatically parcellating the human cerebral cortex. *Cereb Cortex*. 2004;14(1):11-22. doi:10.1093/cercor/bhg087.

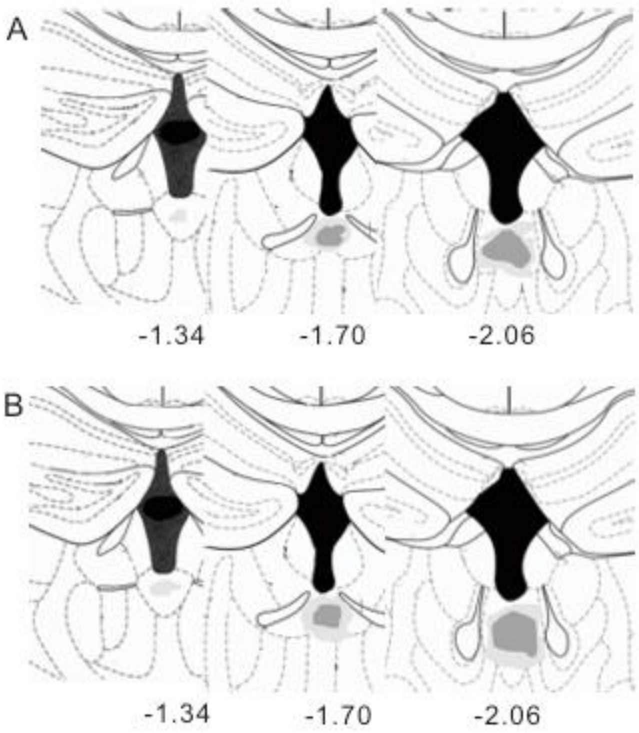


**Supplementary Fig. 1. Schematic representation of bidirectional manipulation of OTR-expressing PVT neurons (related to Fig. 1)**

**(A)** Viral spread validation at the injection sites in the PVT from mice following behavioural testing. Grey areas represent the minimum (lighter colour) and maximum (darker colour) spread of iDREADD within the PVT. **(B)** Viral spread validation at the injection sites in the PVT from mice following behavioural testing. Grey areas represent the minimum (lighter colour) and maximum (darker colour) spread of eDREADD within the PVT.

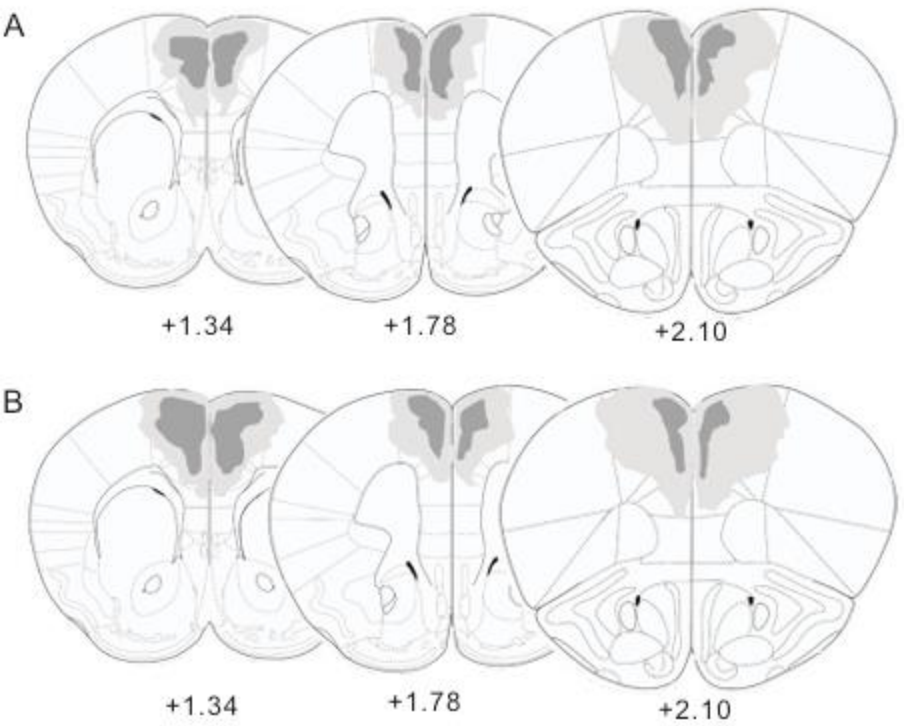


**Supplementary Fig. 2. Schematic representation of bidirectional manipulation of OTR-expressing mPFC neurons (related to Fig. 1)**

**(A)** Viral spread validation at the injection sites in the mPFC from mice following behavioural testing. Grey areas represent the minimum (lighter colour) and maximum (darker colour) spread of iDREADD within the mPFC. **(B)** Viral spread validation at the injection sites in the mPFC from mice following behavioural testing. Grey areas represent the minimum (lighter colour) and maximum (darker colour) spread of eDREADD within the mPFC.

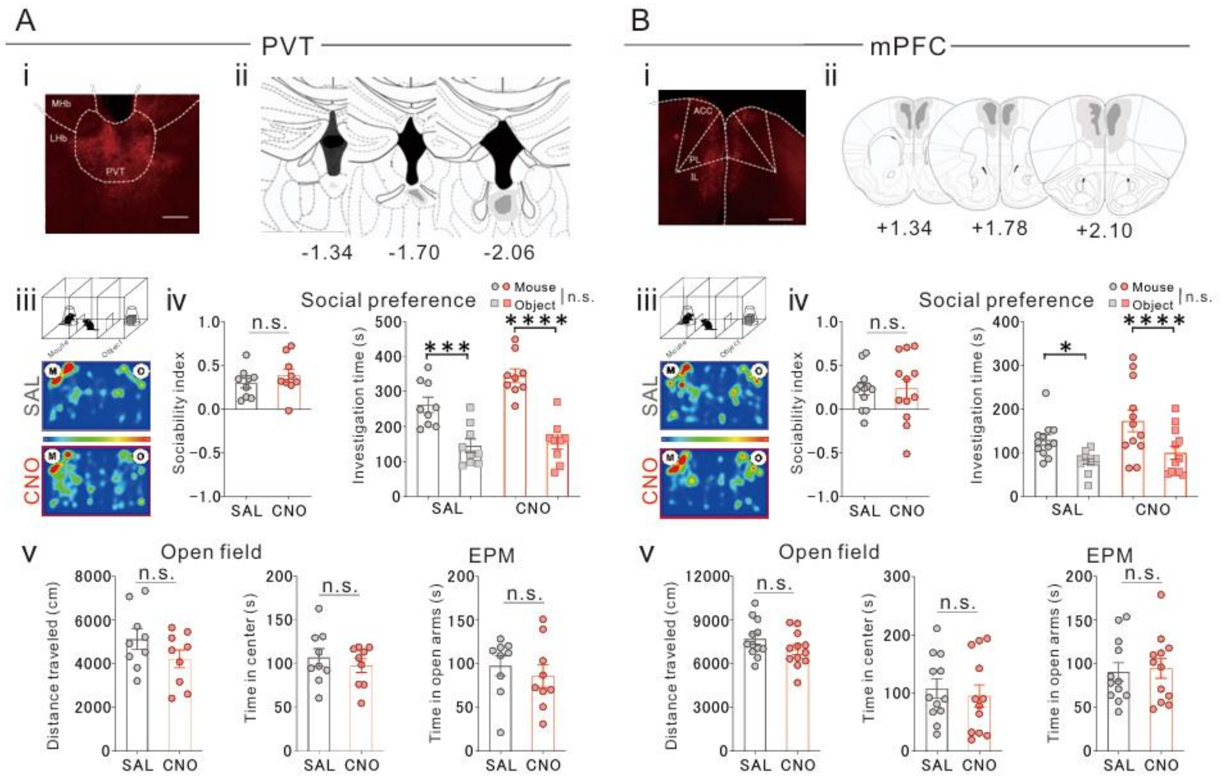


**Supplementary Fig. 3. Chemogenetic control mice show no changes in sociability (related to Fig. 1)**

**(Ai)** Representative PVT images showing mCherry expression. Scale bar: 500 µm. **(Aii)** Viral spread validation at the injection sites in the PVT from mice following behavioural testing. Grey areas represent the minimum (lighter colour) and maximum (darker colour) spread of mCherry within the PVT. **(Aiii)** Cre-dependent mCherry vector was injected into the PVT of *Oxtr*-Cre mice to express mCherry in OTR-expressing neurons. Adult mice received intraperitoneal SAL or CNO (5 mg/kg) before a three-chamber sociability test. **(Aiv)** No sociability differences were observed between CNO- and SAL-treated mCherry-expressing control mice (SAL: n = 9, CNO: n = 9). Left: unpaired two-tailed *t*-test, $t_{16}$ = 0.918, $p$ = 0.3725. Right: two-way RM ANOVA, drug (CNO/SAL) × stimulus (social/object) interaction, $F_{1,32}$ = 1.963, $p$ = 0.1709; drug effect $F_{1,32}$ = 3.822, $p$ = 0.0594; stimulus effect $F_{1,32}$ = 61.770, ****$p$ < 0.0001, Bonferroni post-hoc: social vs.

object in SAL ***$p$ = 0.0001, in CNO ****$p$ < 0.0001. **(Av)** No differences in motor activity or anxiety-related behaviour were observed (open-field distance travelled: $t_{16} = 1.477$, $p = 0.1591$; time in centre: $t_{16} = 0.714$, $p = 0.4855$; open arm time: $t_{16} = 0.676$, $p = 0.5085$; SAL: n = 9, CNO: n = 9). **(Bi)** mPFC images of mCherry. Scale bar: 500 µm. **(Bii)** Viral spread validation at the injection sites in the mPFC from mice following behavioural testing. Grey areas represent the minimum (lighter colour) and maximum (darker colour) spread of mCherry within the mPFC. **(Biii)** A Cre-dependent mCherry vector was injected into the mPFC to express mCherry in OTR-expressing neurons. Adult mice received intraperitoneal SAL or CNO (5 mg/kg) and underwent a three-chamber sociability test. **(Biv)** No sociability differences were observed between CNO- and SAL-treated mCherry-expressing control mice (SAL: n = 12, CNO: n = 12). Left: *t*-test, $t_{22} = 0.031$, $p = 0.9754$. Right: two-way RM ANOVA, drug × stimulus interaction, $F_{1,44} = 2.481$, $P = 0.1224$; drug effect $F_{1,44} = 4.758$, *$p$ = 0.0345; stimulus effect $F_{1,44} = 24.250$, ****$p$ < 0.0001, Bonferroni post-hoc: social vs. object in SAL *$p$ = 0.0441, in CNO ****$p$ < 0.0001. **(Bv)** No differences in motor activity or anxiety-related behaviour were observed (open-field distance travelled, *t*-test, $t_{22} = 1.486$, $p = 0.1514$; time in centre, *t*-test, $t_{22} = 0.511$, $p = 0.6143$; EPM open arm time, $t_{22} = 0.257$, $p = 0.7993$; SAL: n = 12, CNO: n = 12).

*Abbreviations:* EPM, elevated plus maze; RM ANOVA, repeated-measures analysis of variance; OTR, oxytocin receptor; SAL, saline; CNO, clozapine N-oxide dihydrochloride; PVT, paraventricular thalamus; mPFC, medial prefrontal cortex.

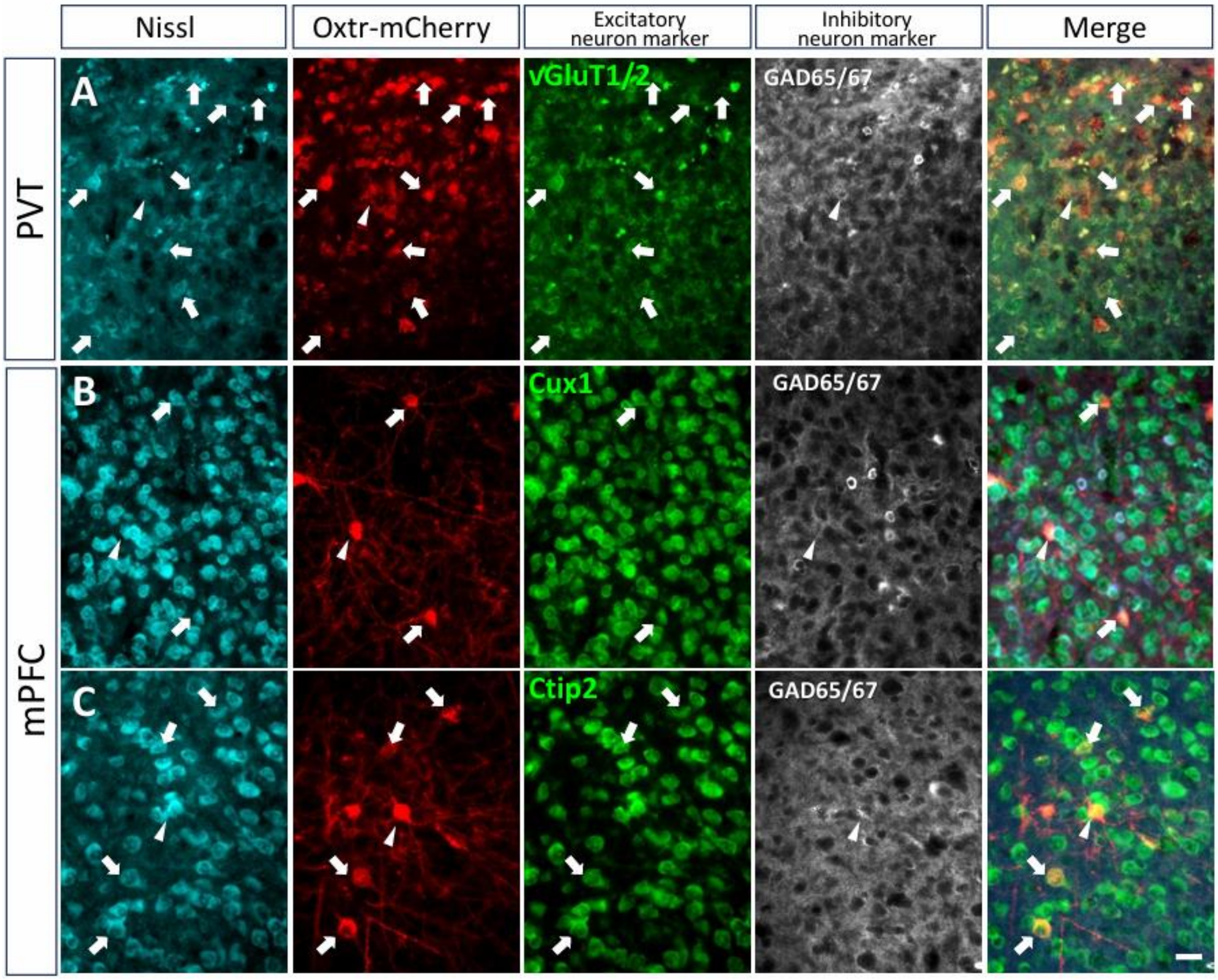


**Supplementary Fig. 4. Cell-type characterisation of OTR-expressing neurons in the PVT and mPFC**

To assess the neuronal identity of the OTR-expressing neurons targeted by chemogenetic manipulation, we performed immunohistochemical co-labelling with excitatory (vGluT1/2, Cux1, Ctip2) and inhibitory (GAD65/67) neuronal markers.

**(A)** In the PVT, a total of 108 mCherry-labelled OTR-expressing neurons were analysed across nine sections from four *Oxtr*-Cre mice. Among these, 104 neurons (96%) co-expressed vGluT1/2, while only four neurons (4%) were GAD65/67+, indicating that OTR-expressing PVT neurons are almost exclusively excitatory.

**(B)** In the mPFC, we analysed 50 OTR-expressing neurons across six sections from four mice using Cux1 and GAD65/67. Of these, 37 neurons (74%) were Cux1+ and 13 neurons (26%) were GAD65/67+.

**(C)** In a separate mPFC dataset (51 OTR-expressing neurons from four mice), co-labelling with Ctip2 and GAD65/67 revealed that 38 neurons (75%) were Ctip2+ and 13 neurons (25%) were GAD65/67+. These results indicate that while OTR-expressing neurons are predominantly excitatory in both regions, the mPFC contains a consistent inhibitory subpopulation (~25%).

*Abbreviations:* OTR, oxytocin receptor; PVT, paraventricular thalamus; mPFC, medial prefrontal cortex.

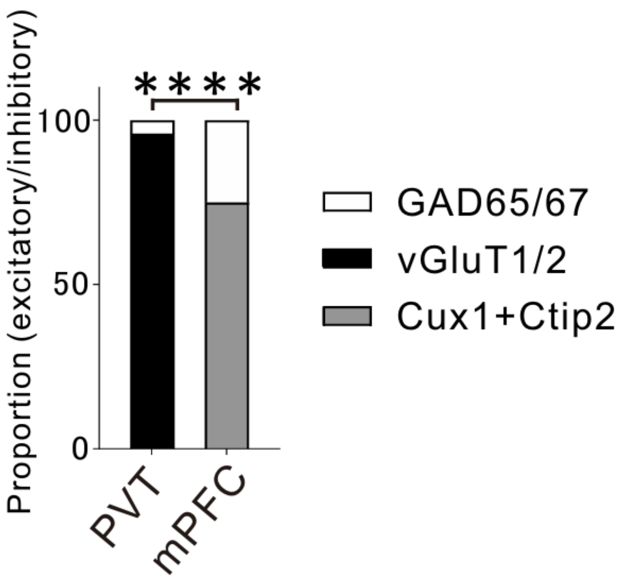


**Supplementary Fig. 5. Regional difference in the neuronal subtype composition of OTR-expressing cells between the PVT and mPFC**

**(A)** A chi-square test comparing the proportion of excitatory and inhibitory OTR-expressing neurons between the PVT (104 excitatory, 4 inhibitory) and combined mPFC datasets (75 excitatory, 26 inhibitory) revealed a statistically significant difference in neuronal subtype distribution ($\chi^2 = 18.867$, df = 1, ****$p < 0.0001$).

*Abbreviations:* OTR, oxytocin receptor; PVT, paraventricular thalamus; mPFC, medial prefrontal cortex.

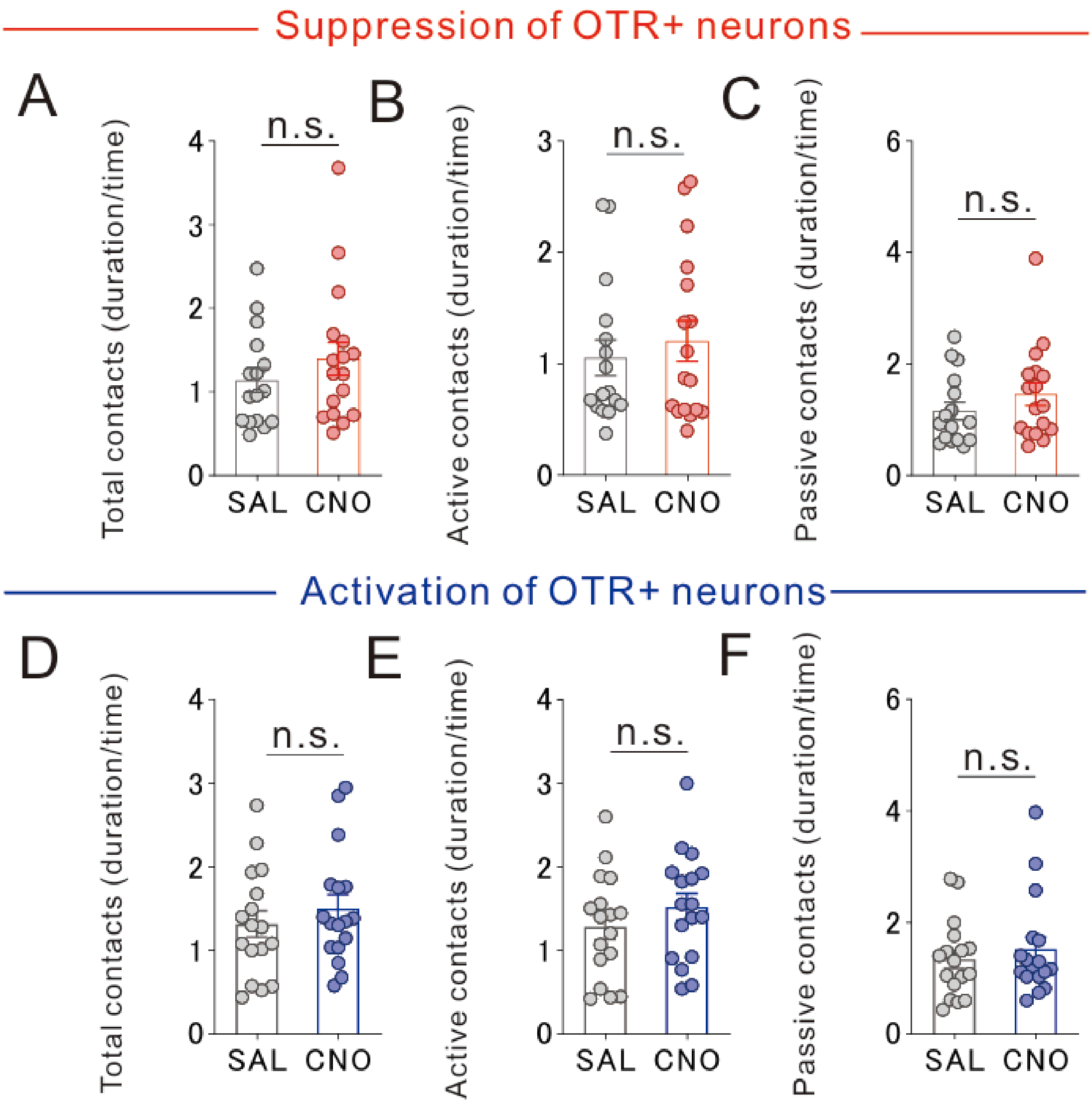


**Supplementary Fig. 6. Contact duration for individual interactions in free-moving conditions (related to Fig. 2)**

Mice were allowed to interact freely for 60 min with three unfamiliar cage-mates in a novel environment. **(A)** CNO-treated iDREADD+ mice showed no difference in total contact duration per contact compared to SAL-treated mice (Mann–Whitney *U* test, $U = 109$, $p = 0.3445$; SAL: n = 16, CNO: n = 17).

**(B)** CNO-treated iDREADD+ mice showed no difference in active contact duration per contact compared to SAL-treated mice (Mann–Whitney *U*-test, $U = 131$, $p = 0.8730$; SAL: n = 16, CNO: n = 17).

**(C)** CNO-treated iDREADD+ mice showed no difference in passive contact duration per contact compared to SAL-treated mice (Mann–Whitney *U*-test, $U = 106$, $p = 0.2922$; SAL: n = 16, CNO: n = 17).

**(D)** CNO-treated eDREADD+ mice showed no difference in total contact duration per contact compared to SAL-treated mice (unpaired two-tailed *t*-test, $t_{32} = 0.824$, $p = 0.4159$; SAL: n = 17, CNO: n = 17).

**(E)** CNO-treated eDREADD+ mice showed no difference in active contact duration per contact compared to SAL-treated mice (unpaired two-tailed *t*-test, $t_{32} = 1.087$, $p = 0.2851$; SAL: n = 17, CNO: n = 17).

**(F)** CNO-treated eDREADD+ mice showed no difference in passive contact duration per contact compared to SAL-treated mice (Mann–Whitney *U*-test, $U = 134.5$, $p = 0.7405$; SAL: n = 17, CNO: n = 17).

*Abbreviations:* iDREADD, inhibitory designer receptors exclusively activated by designer drugs; eDREADD, excitatory DREADD; SAL, saline; CNO, clozapine N-oxide dihydrochloride.

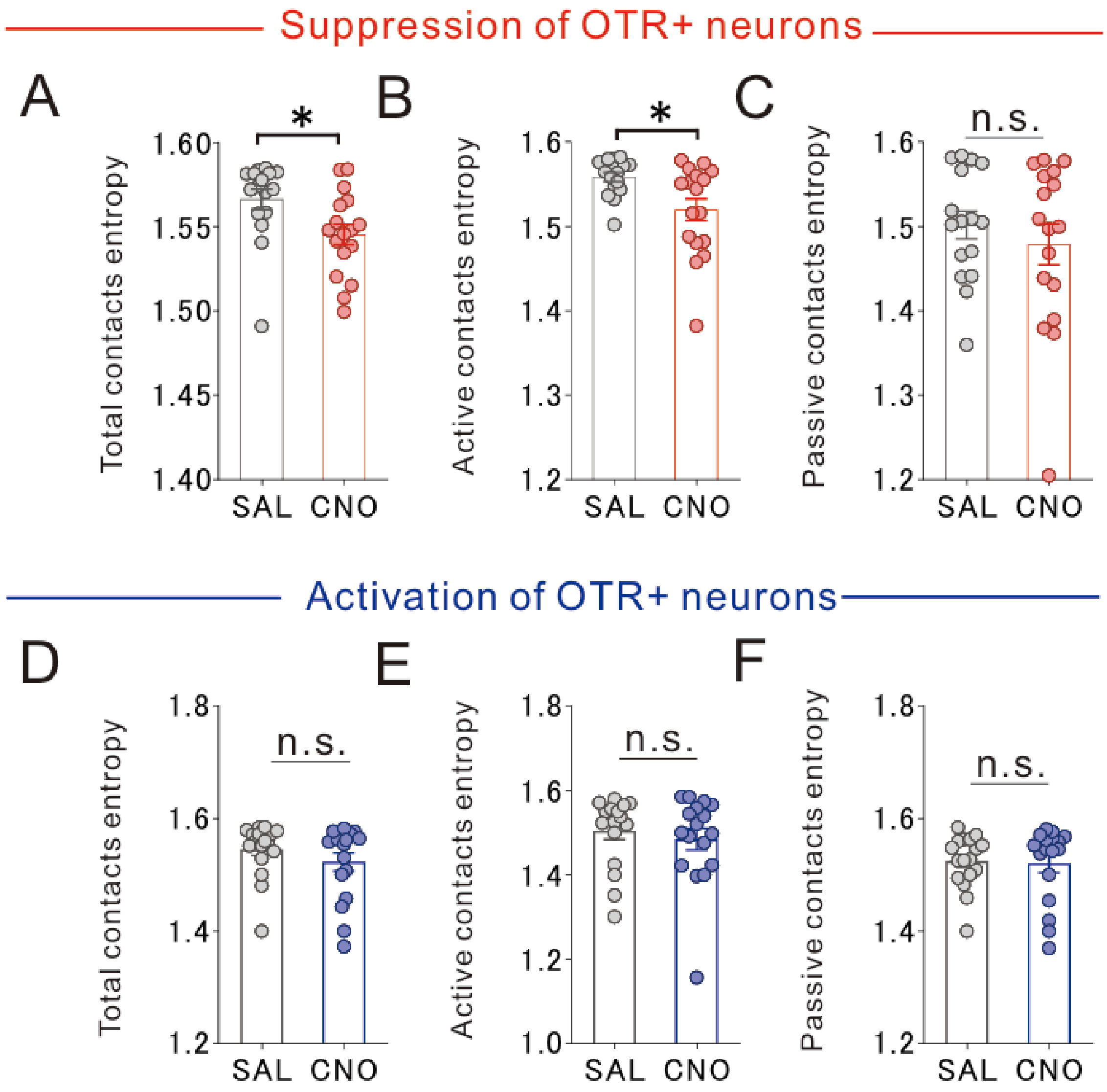


**Supplementary Fig. 7. Entropy in free-moving conditions (related to Fig. 2)**

**(A)** CNO-treated iDREADD+ mice showed reduced entropy in total contact behaviour compared to SAL-treated mice (Mann–Whitney *U* test, $U = 68$, $*p = 0.0136$; SAL: n = 16, CNO: n = 17).

**(B)** CNO-treated iDREADD+ mice showed reduced entropy in active contact behaviour compared to SAL-treated mice (Mann–Whitney *U* test, $U = 72$, $*p = 0.0207$; SAL: n = 16, CNO: n = 17).

**(C)** CNO-treated iDREADD+ mice showed no difference in entropy in passive contact behaviour compared to SAL-treated mice (Mann–Whitney $U$ test, $U = 115$, $p = 0.4654$; SAL: n = 16, CNO: n = 17).

**(D)** CNO-treated eDREADD+ mice showed no difference in entropy in total contact behaviour compared to SAL-treated mice (Mann–Whitney $U$ test, $U = 120$, $p = 0.4084$; SAL: n = 17, CNO: n = 17).

**(E)** CNO-treated eDREADD+ mice showed no difference entropy in active contact behaviour compared to SAL-treated mice (Mann–Whitney $U$ test, $U = 129$, $p = 0.6039$; SAL: n = 17, CNO: n = 17).

**(F)** CNO-treated eDREADD+ mice showed no difference in entropy in passive contact behaviour compared to SAL-treated mice (Mann–Whitney $U$ test, $U = 133$, $p = 0.7021$; SAL: n = 17, CNO: n = 17).

*Abbreviations:* iDREADD, inhibitory designer receptors exclusively activated by designer drugs; eDREADD, excitatory DREADD; SAL, saline; CNO, clozapine N-oxide dihydrochloride

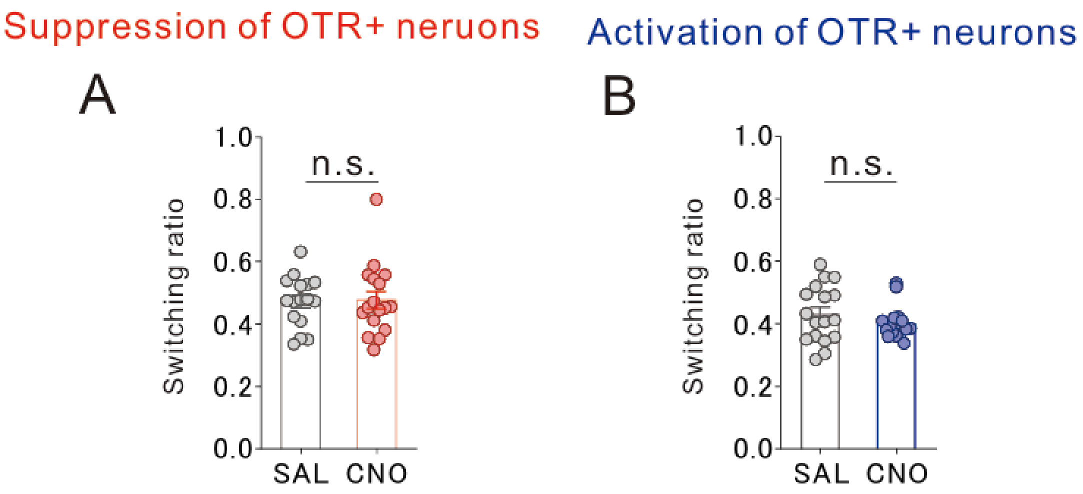


**Supplementary Fig. 8. Switching ratio in free-moving conditions (related to Fig. 2)**

**(A)** CNO-treated iDREADD+ mice showed no difference in switching ratio compared to SAL-treated mice (Mann–Whitney *U* test, $U = 127$, $p = 0.7626$; SAL: n = 16, CNO: n = 17).

**(B)** CNO-treated eDREADD+ mice showed no difference in switching ratio compared to SAL-treated mice (Mann–Whitney *U* test, $U = 121$, $p = 0.4332$; SAL: n = 17, CNO: n = 17).

*Abbreviations:* iDREADD, inhibitory designer receptors exclusively activated by designer drugs; eDREADD, excitatory DREADD; SAL, saline; CNO, clozapine N-oxide dihydrochloride.

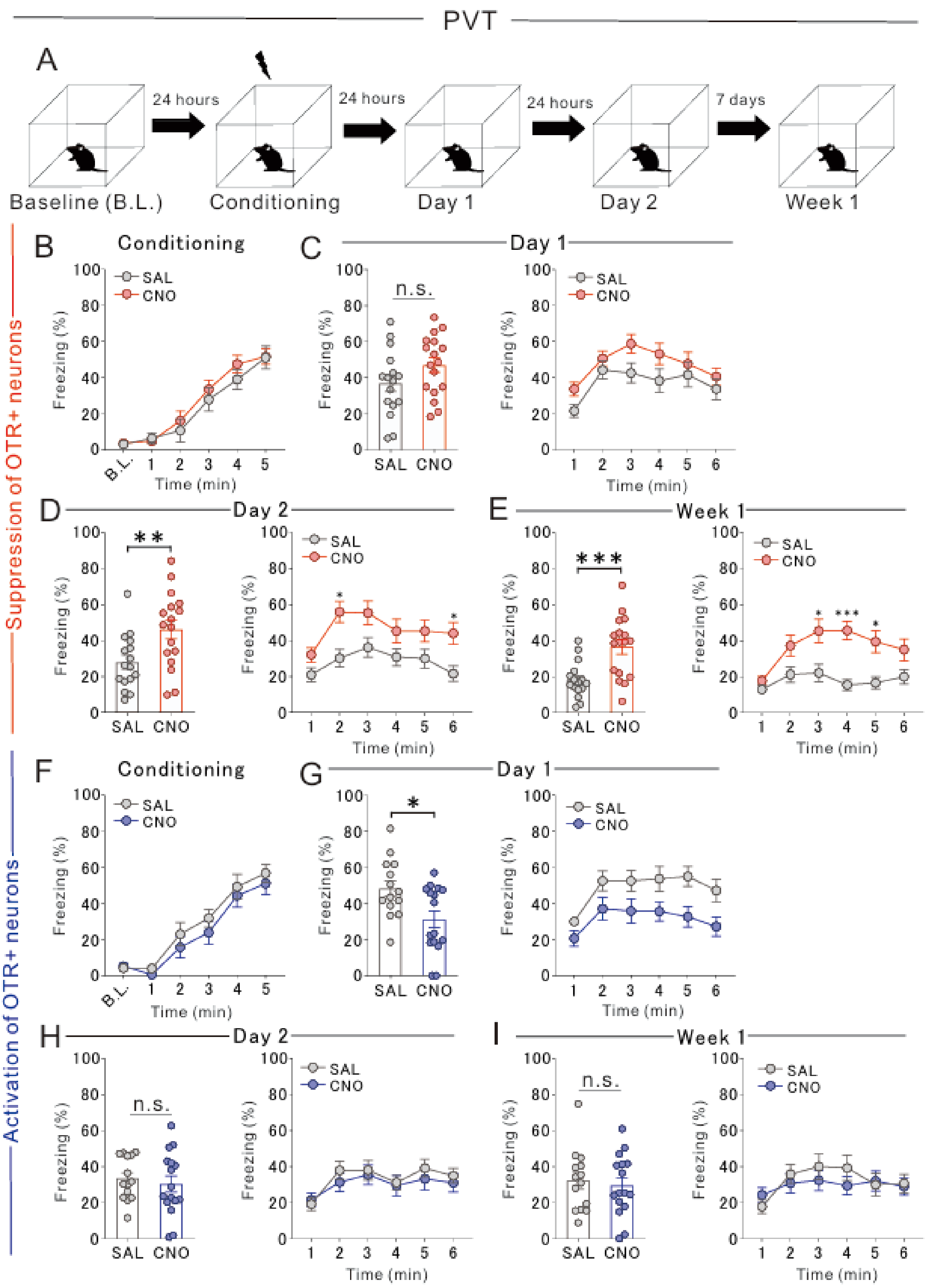
PVT
A
24 hours
24 hours
24 hours
7 days
Baseline (B.L.)
Conditioning
Day 1
Day 2
Week 1
Suppression of OTR+ neurons
B
Conditioning
SAL
CNO
Freezing (%)
Time (min)
C
Day 1
n.s.
D
Day 2
E
Week 1
Activation of OTR+ neurons
F
Conditioning
G
Day 1
H
Day 2
I
Week 1

1

**Supplementary Fig. 9. Chemogenetic modulation of OTR-expressing PVT neurons affects contextual fear extinction**

**(A)** Schematic representation of the behavioural design. Cre-dependent iDREADD/eDREADD vectors were injected into the PVT of *Oxtr*-Cre mice. SAL or CNO (iDREADD: 5 mg/kg, eDREADD: 1 mg/kg) was administered 30 min prior to behavioural testing on Day 1, Day 2, and Week 1. (SAL: n = 16, CNO: n = 17 for iDREADD and SAL: n = 14, CNO: n = 16 for eDREADD)

**(B)** CNO-treated iDREADD+ mice showed no difference in fear conditioning vs. SAL-treated mice (two-way RM ANOVA, drug (CNO/SAL) × time interaction, $F_{5,155} = 0.536$, $p = 0.7492$; drug effect $F_{1,31} = 0.535$, $p = 0.4699$; time effect $F_{3.210,99.50} = 57.370$, ****$p < 0.0001$).

**(C)** No difference in freezing ratio on Day 1. Left: unpaired *t*-test, $t_{31} = 1.669$, $p = 0.1053$. Right: two-way RM ANOVA, drug × time interaction, $F_{5,155} = 0.843$, $p = 0.5214$; drug effect $F_{1,31} = 2.885$, $p = 0.0994$; time effect $F_{3.121, 96.76} = 11.570$, ****$p < 0.0001$.

**(D)** Increased freezing on Day 2 in CNO-treated iDREADD+ mice. Left: unpaired *t*-test, $t_{31} = 2.836$, **$p = 0.0080$. Right: two-way RM ANOVA, drug × time interaction, $F_{5,155} = 1.460$, $p = 0.2060$; drug effect $F_{1,31} = 7.794$, **$p = 0.0089$; time effect $F_{4.109, 127.4} = 9.680$, ****$p < 0.0001$, Bonferroni post-hoc: SAL vs. CNO in session 2 *$p = 0.0140$, SAL vs. CNO in session 6 ***$p = 0.0310$.

**(E)** Increased freezing at Week 1 in CNO-treated iDREADD+ mice. Left: Welch's *t*-test, $t_{26.25} = 3.863$, ***$p = 0.0007$. Right: two-way RM ANOVA, drug × time interaction, $F_{5,155} = 3.038$, *$p = 0.0121$; drug effect $F_{1,31} = 14.760$, ***$p = 0.0006$; time effect $F_{3.576,110.8} = 6.287$, ***$p = 0.0002$,

Bonferroni post-hoc: SAL vs. CNO in session 3 *$p = 0.0388$, SAL vs. CNO in session 4 ***$p = 0.0001$, SAL vs. CNO in session 6 *$p = 0.0164$.

**(F)** CNO-treated eDREADD+ mice showed no difference in fear conditioning compared to SAL (two-way RM ANOVA, drug × time interaction, $F_{5,155} = 0.536$, $p = 0.7492$; drug effect $F_{1,31} = 0.535$, $p = 0.4699$; time effect $F_{3.210,99.50} = 557.370$, ****$p < 0.0001$; SAL: n = 14, CNO: n = 16).

**(G)** Reduced freezing on Day 1 in CNO-treated eDREADD+ mice. Left: unpaired *t*-test, $t_{28} = 2.836$, *$p = 0.0122$. Right: two-way RM ANOVA, drug × time interaction, $F_{5,140} = 0.730$, $p = 0.6021$; drug effect $F_{1,28} = 6.986$, *$p = 0.0133$; time effect $F_{3.940,\ 110.3} = 8.628$, ****$p < 0.0001$.

**(H)** No difference in freezing on Day 2. Left: unpaired *t*-test, $t_{28} = 0.553$, $p = 0.5847$. Right: two-way RM ANOVA, drug × time interaction, $F_{5,140} = 0.465$, $p = 0.8016$; drug effect $F_{1,28} = 0.299$, $p = 0.5891$; time effect $F_{4.462,\ 124.9} = 6.540$, ****$p < 0.0001$).

**(I)** No difference in freezing at Week 1. Left: unpaired *t*-test, $t_{28} = 0.412$, $p = 0.6834$. Right: two-way RM ANOVA, drug × time interaction, $F_{5,140} = 1.576$, $p = 0.1706$; drug effect $F_{1,28} = 0.175$, $p = 0.6793$; time effect $F_{4.097,\ 114.7} = 4.846$, **$p = 0.0011$.

*Abbreviations:* iDREADD, inhibitory designer receptors exclusively activated by designer drugs; eDREADD, excitatory DREADD; SAL, saline; CNO, clozapine N-oxide dihydrochloride; RM ANOVA, repeated-measures analysis of variance; PVT, paraventricular thalamus; OTR, oxytocin receptor.

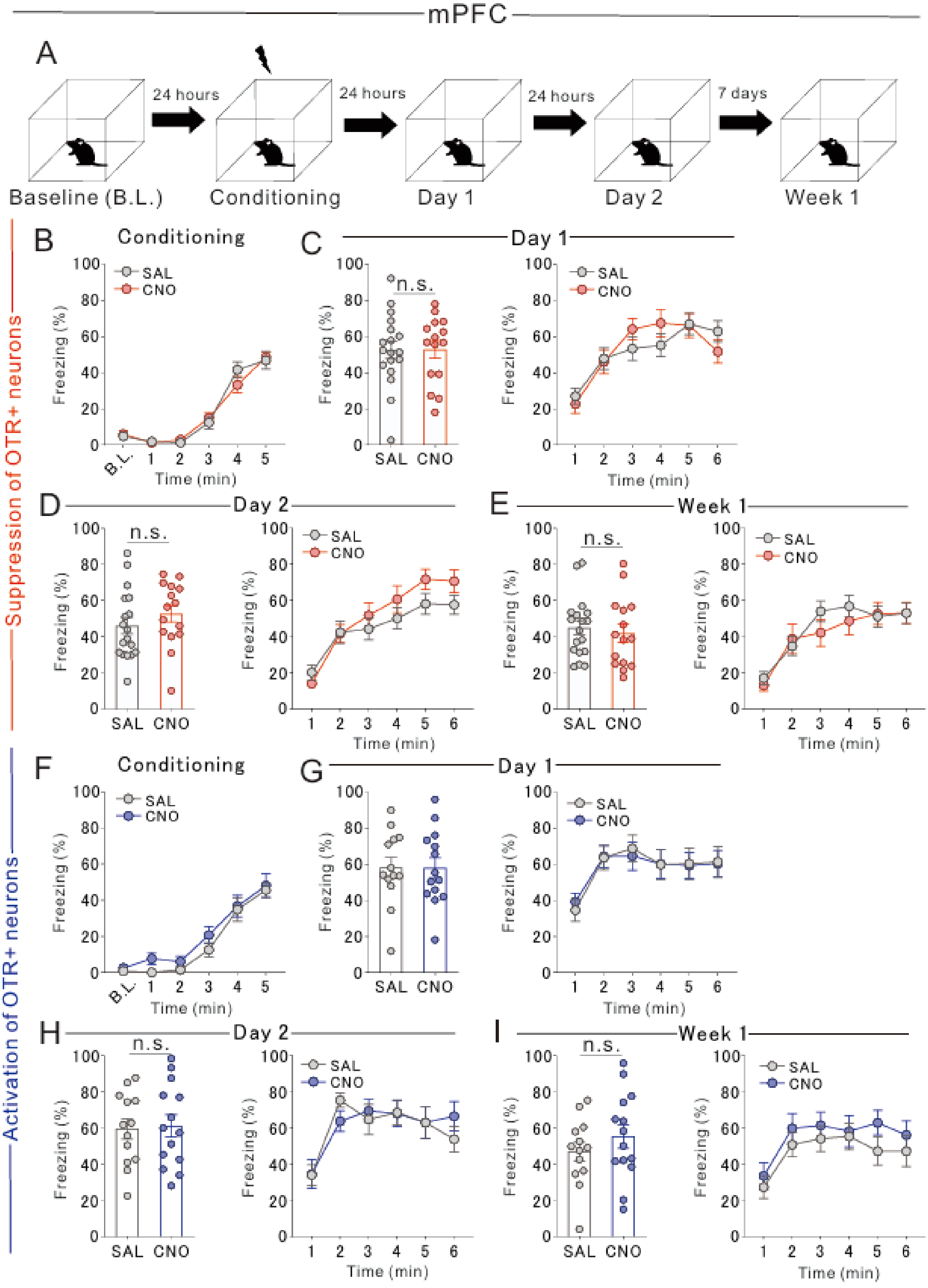
mPFC
A
24 hours
24 hours
24 hours
7 days
Baseline (B.L.)
Conditioning
Day 1
Day 2
Week 1
Suppression of OTR+ neurons
B
Conditioning
C
Day 1
D
Day 2
E
Week 1
SAL
CNO
n.s.
Freezing (%)
Time (min)
Activation of OTR+ neurons
F
Conditioning
G
Day 1
H
Day 2
I
Week 1

**Supplementary Fig. 10. Chemogenetic modulation of the mPFC does not affect contextual fear extinction learning**

**(A)** Schematic representation of the behavioural design. Cre-dependent iDREADD/eDREADD vectors were injected into the mPFC of Oxtr-Cre mice. SAL or CNO (iDREADD+ mice: 5 mg/kg CNO; eDREADD+ mice: 1 mg/kg CNO) was administered 30 min prior to experiments on Day 1, Day 2, and Week 1. (SAL: n = 18, CNO: n = 15 for iDREADD and SAL: n = 13, CNO: n = 14 for eDREADD)

**(B)** CNO-treated iDREADD+ mice showed no significant differences in freezing behaviour during fear conditioning vs. SAL-treated controls (two-way RM ANOVA, drug (CNO/SAL) × time interaction, $F_{5,155} = 0.955$, $p = 0.4473$; drug effect $F_{1,31} = 0.039$, $p = 0.8453$; time effect $F_{3.116,95.58} = 91.46$, ****$p < 0.0001$).

**(C)** No difference in freezing ratio on Day 1. Left: unpaired *t*-test, $t_{31} = 0.025$, $p = 0.9805$. Right: two-way RM ANOVA, drug × time interaction, $F_{5,155} = 2.349$, *$p = 0.0435$; drug effect $F_{1,31} = 0.0136$, $p = 0.9078$; time effect $F_{3.927, 121.7} = 25.920$, ****$p < 0.0001$.

**(D)** No difference in freezing ratio on Day 2. Left: unpaired *t*-test, $t_{31} = 1.021$, $p = 0.3151$. Right: two-way RM ANOVA, drug × time interaction, $F_{5,155} = 2.304$, *$p = 0.0472$; drug effect $F_{1,31} = 0.932$, $p = 0.3418$; time effect $F_{3.541, 109.8} = 45.670$, ****$p < 0.0001$.

**(E)** No difference in freezing ratio at Week 1. Left: unpaired *t*-test, $t_{31} = 0.460$, $p = 0.6487$. Right: two-way RM ANOVA, drug× time interaction, $F_{5,155} = 1.019$, *$p = 0.4082$; drug effect $F_{1,31} = 0.229$, $P = 0.6356$; time effect $F_{3.623, 112.3} = 24.800$, ****$p < 0.0001$.

**(F)** No difference in fear conditioning for CNO-treated eDREADD+ mice vs. SAL (two-way RM ANOVA, drug × time interaction, $F_{5,125} = 0.266$, $p = 0.9307$; drug effect $F_{1,25} = 3.182$, $p = 0.0866$; time effect $F_{2.901,72.53} = 45.00$, $^{****}p < 0.0001$).

**(G)** No difference in freezing ratio on Day 1. Left: unpaired *t*-test, $t_{25} = 0.018$, $p = 0.9857$. Right: two-way RM ANOVA, drug × time interaction, $F_{5,125} = 0.161$, $p = 0.9764$; drug effect $F_{1,25} = 0.001$, $p = 0.9903$; time effect $F_{3.458, 86.46} = 8.687$, $^{****}p < 0.0001$.

**(H)** No difference in freezing ratio on Day 2. Left: unpaired *t*-test, $t_{25} = 0.191$, $p = 0.8503$. Right: two-way RM ANOVA, drug × time interaction, $F_{5,125} = 1.438$, $p = 0.2153$; drug effect $F_{1,25} = 0.014$, $p = 0.9073$; time effect $F_{3.783, 94.58} = 16.380$, $^{****}p < 0.0001$).

**(I)** No difference in freezing ratio at Week 1. Left: unpaired *t*-test, $t_{25} = 0.997$, $p = 0.3282$. Right: two-way RM ANOVA, drug × time interaction, $F_{5,125} = 0.337$, $p = 0.8896$; drug effect $F_{1,25} = 0.991$, $p = 0.3291$; time effect $F_{3.361, 84.03} = 8.000$, $^{****}p < 0.0001$).

*Abbreviations:* iDREADD, inhibitory designer receptors exclusively activated by designer drugs; eDREADD, excitatory DREADD; SAL, saline; CNO, clozapine N-oxide dihydrochloride; RM ANOVA, repeated-measures analysis of variance; mPFC, medial prefrontal cortex.

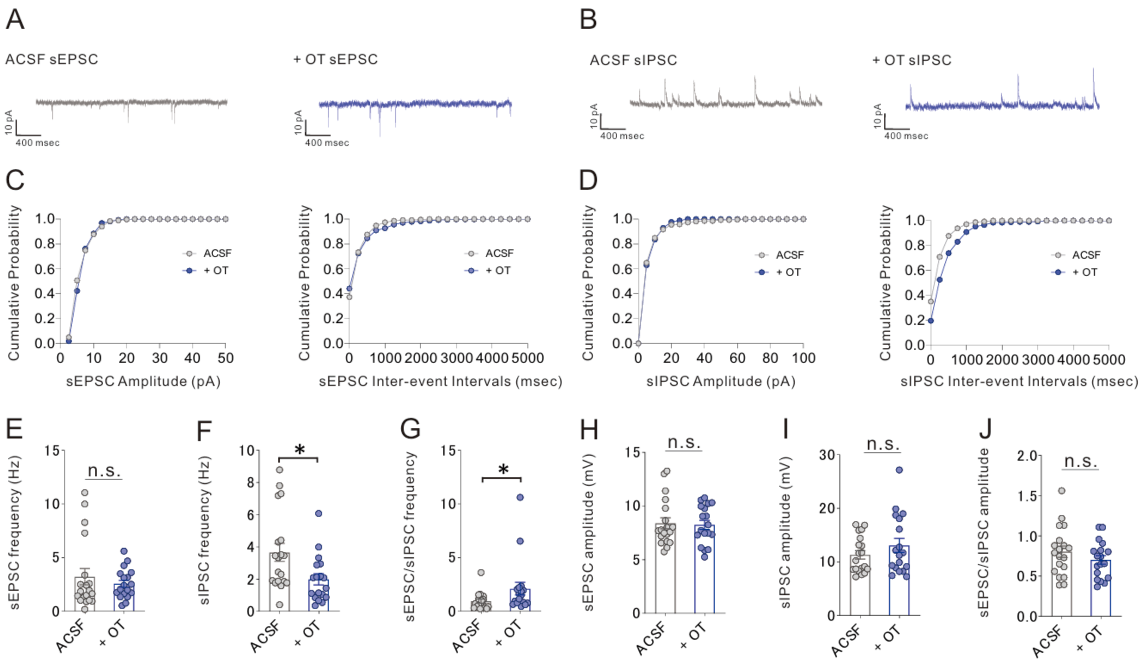


**Supplementary Fig. 11. Oxytocin modulates excitatory/inhibitory inputs to OTR-expressing PVT neurons (related to Fig. 5)**

These electrophysiological recordings were conducted in mCherry-labelled OTR+ neurons.

**(A)** Representative traces of sEPSCs recorded from OTR-expressing PVT neurons under normal ACSF conditions (left) and oxytocin-added ACSF (+OT) conditions (right).

**(B)** Representative traces of sIPSCs recorded from OTR-expressing PVT neurons under normal ACSF conditions (left) and oxytocin-added ACSF (+OT) conditions (right).

**(C)** Cumulative probability plots of sEPSC amplitudes (left) and inter-event intervals (right) under ACSF and +OT conditions.

**(D)** Cumulative probability plots of sIPSC amplitudes (left) and inter-event intervals (right) under ACSF and +OT conditions.

**(E)** No difference in sEPSC frequency between normal ACSF and +OT conditions in OTR-expressing PVT neurons of Oxtr-Cre adult mice (Mann–Whitney U test, U=151.5, p=0.5628; normal ACSF: n=19, +OT: n=18).

**(F)** Oxytocin decreases sIPSC frequency in +OT vs. normal ACSF in OTR-expressing PVT neurons (Mann–Whitney U test, U=91.5, *p=0.0147; normal ACSF: n=19, +OT: n=18).

**(G)** Oxytocin increases sEPSC/sIPSC frequency ratio in +OT vs. normal ACSF in OTR-expressing PVT neurons (Mann–Whitney U test, U=92, *p=0.0158; normal ACSF: n=19, +OT: n=18).

**(H)** No difference in sEPSC amplitude between normal ACSF and +OT conditions in OTR-expressing PVT neurons (Mann–Whitney U test, U=170, p=0.9880; normal ACSF: n=19, +OT: n=18).

**(I)** No difference in sIPSC amplitude between normal ACSF and +OT conditions in OTR-expressing PVT neurons (Mann–Whitney U test, U=142, p=0.3909; normal ACSF: n=19, +OT: n=18).

**(J)** No difference in the sEPSC/sIPSC amplitude ratio between normal ACSF and +OT conditions in OTR-expressing PVT neurons (unpaired t-test, $t_{35}$= 1.149, p=0.2583; normal ACSF: n=19, +OT: n=18).

*Abbreviations:* sEPSCs, spontaneous excitatory postsynaptic currents; sIPSCs, spontaneous inhibitory postsynaptic currents; OT, oxytocin; OTR, oxytocin receptor; ACSF, artificial cerebrospinal fluid; PVT, paraventricular thalamus.

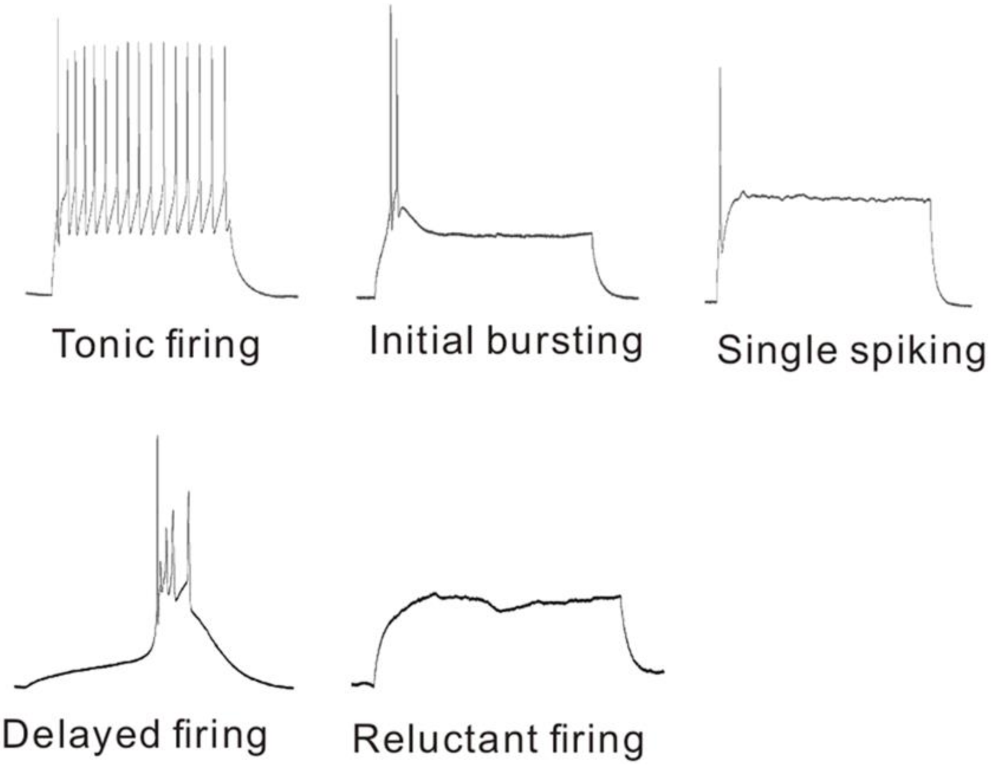


**Supplementary Fig. 12. Firing patterns of PVT neurons**

Five distinct patterns of action potential discharge were observed in PVT neurons in response to increasing amplitudes of current step injection: tonic firing, initial bursting, single spiking, delayed firing, and reluctant firing.

*Abbreviations*: PVT, paraventricular thalamus.

**Supplementary Table 1. Participant background**

| Dependent variable and covariate | TD | | ASD | | | |
|---|---|---|---|---|---|---|
| | **average** | **SD** | **average** | **SD** | ***t*-value** | ***p*-value** |
| Age (years) | 14.270 | 1.348 | 14.526 | 1.264 | −0.517 | 0.609 |
| Sex (male/female) | 8/3 | | 15/4 | | | 0.868 |
| Oxytocin | 20.931 | 13.242 | 18.157 | 17.345 | 0.458 | 0.651 |
| NDI thalamus | 0.548 | 0.029 | 0.558 | 0.020 | −1.114 | 0.550 |
| NDI DLPFC | 0.407 | 0.006 | 0.413 | 0.012 | −0.156 | 0.260 |
| ODI thalamus | 0.331 | 0.011 | 0.333 | 0.009 | −0.624 | 1.000 |
| ODI DLPFC | 0.501 | 0.010 | 0.498 | 0.014 | −0.568 | 1.000 |
| AQ-J total | 16.091 | 6.057 | 27.895 | 6.927 | −4.699 | **<0.001** |
| AQ-J social skill | 3.182 | 2.228 | 6.842 | 2.522 | −3.990 | **<0.001** |
| AQ-J attention switching | 3.636 | 1.567 | 5.789 | 1.843 | −3.248 | **0.003** |
| AQ-J local detail | 4.182 | 2.401 | 5.105 | 2.183 | −1.077 | 1.000 |
| AQ-J communication | 2.364 | 1.502 | 5.053 | 2.460 | −3.275 | **0.018** |
| AQ-J imagination | 2.727 | 2.005 | 5.053 | 2.592 | −2.559 | 0.096 |
| GHQ-30 general disease trends | 0.636 | 0.809 | 1.632 | 1.212 | −2.421 | 0.132 |
| GHQ-30 physical conditions | 1.364 | 1.120 | 1.684 | 1.455 | −0.629 | 1.000 |
| GHQ-30 sleeping disorder | 0.364 | 0.674 | 2.263 | 1.695 | −4.330 | **0.006** |
| GHQ-30 social activity disorder | 0.273 | 0.647 | 1.579 | 1.305 | −3.092 | **0.024** |
| GHQ-30 anxiety and dysthymia | 0.273 | 0.467 | 2.263 | 1.759 | −3.654 | **0.006** |
| GHQ-30 suicidal ideation and depression | 0.091 | 0.302 | 2.000 | 2.211 | −2.828 | **0.006** |

TD, typically developing; ASD, autism-spectrum disorder; NDI, neurite density index; ODI, orientation dispersion index; AQ-J, Japanese version of the Autism-Spectrum Quotient; GHQ-30, a 30-item General Health Questionnaire; ADOS-2, Autism Diagnostic Observation Schedule, second edition; SD, standard deviation; DLPFC, dorsolateral prefrontal cortex

**Supplementary Table 2.** Multiple regression analysis of variables associated with the oxytocin in patients with ASD and TD individuals

| Dependent variable and covariate | B | SE | β | *t*-value | *p*-value | $R^2$ | Adjusted $R^2$ | *p*-value |
|---|---|---|---|---|---|---|---|---|
| Oxytocin | | | | | | 0.200 | 0.141 | **0.049** |
| NDI thalamus | 290.390 | 121.056 | 0.434 | 2.399 | **0.024** | | | |
| NDI DLPFC | 58.976 | 273.732 | 0.039 | 0.215 | 0.831 | | | |
| Oxytocin | | | | | | 0.124 | 0.059 | 0.169 |

ASD, autism-spectrum disorder; TD, typically developing; NDI, neurite density index; SE, standard error; DLPFC, dorsolateral prefrontal cortex

**Supplementary Table 3.** Multiple regression analysis of variables associated with the ODI in patients with ASD and TD individuals

| Dependent variable and covariate | $R^2$ | Adjusted $R^2$ | *p*-value |
|---|---|---|---|
| AQ-J total | 0.071 | −0.036 | 0.582 |
| AQ-J social skill | 0.076 | –0.030 | 0.551 |
| AQ-J attention switching | 0.054 | −0.055 | 0.690 |
| AQ-J local detail | 0.108 | 0.006 | 0.385 |
| AQ-J communication | 0.171 | 0.075 | 0.174 |
| AQ-J imagination | 0.181 | 0.086 | 0.152 |
| GHQ-30 general disease trends | 0.148 | 0.049 | 0.237 |
| GHQ-30 physical conditions | 0.029 | −0.083 | 0.854 |
| GHQ-30 sleeping disorder | 0.032 | −0.079 | 0.833 |
| GHQ-30 social activity disorder | 0.076 | −0.031 | 0.533 |
| GHQ-30 anxiety and dysthymia | 0.060 | −0.049 | 0.651 |
| GHQ-30 suicidal ideation and depression | 0.125 | 0.024 | 0.315 |

ASD, autism-spectrum disorder; TD, typically developing; ODI, orientation dispersion index; AQ-J, Japanese version of the Autism-Spectrum Quotient; GHQ-30, a 30-item General Health Questionnaire

**Supplementary Table 4.** Correlation between salivary oxytocin levels and brain microstructural indices in the combined sample (TD + ASD)

| Dependent variable and covariate | r | *p*-value |
|---|---|---|
| NDI thalamus | 0.006 | 0.970 |
| NDI DLPFC | −0.129 | 0.440 |
| ODI thalamus | 0.190 | 0.253 |
| ODI DLPFC | −0.251 | 0.128 |

ASD, autism-spectrum disorder; TD, typically developing; NDI, neurite density index; ODI, orientation dispersion index; DLPFC, dorsolateral prefrontal cortex

**Supplementary Table 5.** Correlation between salivary oxytocin levels and brain microstructural indices in the TD group

| **Dependent variable and covariate** | **r** | ***p*-value** |
| --- | --- | --- |
| NDI thalamus | 0.829 | 0.006 |
| NDI DLPFC | 0.447 | 0.228 |
| ODI thalamus | 0.681 | 0.043 |
| ODI DLPFC | 0.105 | 0.788 |

TD, typically developing; NDI, neurite density index; ODI, orientation dispersion index; DLPFC, dorsolateral prefrontal cortex

**Supplementary Table 6.** Correlation between salivary oxytocin levels and brain microstructural indices in the ASD group

| Dependent variable and covariate | r | *p*-value |
|---|---|---|
| NDI thalamus | 0.314 | 0.165 |
| NDI DLPFC | 0.281 | 0.217 |
| ODI thalamus | 0.193 | 0.403 |
| ODI DLPFC | −0.270 | 0.236 |

ASD, autism-spectrum disorder; NDI, neurite density index; ODI, orientation dispersion index; DLPFC, dorsolateral prefrontal cortex
